# Symbiont‐mediated chemical defense in the invasive ladybird *Harmonia axyridis*

**DOI:** 10.1002/ece3.4840

**Published:** 2019-01-25

**Authors:** Henrike Schmidtberg, Shantanu P. Shukla, Rayko Halitschke, Heiko Vogel, Andreas Vilcinskas

**Affiliations:** ^1^ Institute for Insect Biotechnology Justus‐Liebig‐University of Giessen Giessen Germany; ^2^ Entomology Department Max‐Planck Institute for Chemical Ecology Jena Germany; ^3^ Department of Bioresources Fraunhofer Institute for Molecular Biology and Applied Ecology Giessen Germany

**Keywords:** chemical defense, gut microbiota, *Harmonia axyridis*, *Lactococcus lactis*, methoxypyrazines (MPs), symbiosis

## Abstract

The volatile alkylpyrazines methyl‐ and methoxypyrazines (MPs) present in the reflex bleeds of coccinellid beetles such as the harlequin ladybird beetle *Harmonia axyridis* are important semiochemicals that function in antipredatory defense behavior. Pyrazines have also been coadapted from a primarily defensive role into pheromones that function in intraspecific communication, attraction, and aggregation behavior. However, the biosynthesis of MPs in ladybird beetles is poorly understood. Here, we tested the hypothesis that MPs could be produced by microbial symbionts in *H. axyridis*, which generates four different MPs. The evaluation of tissue‐specific MP production showed that MP concentrations were highest in the gut tissue and hemolymph of the beetles rather than the fat body tissue as the presumed site of MP biosynthesis. Furthermore, manipulation of gut microbiota by antibiotic‐containing diets resulted in a lower MP content in adult beetles. The analysis of the bacterial community of the digestive tract revealed the presence of bacteria of the genera *Serratia* and *Lactococcus* which are reportedly able to produce MPs. In line with the known diet‐dependent production of MP in *H. axyridis,* we determined that the presence or relative abundance of some of the potential MP producers (*Enterococcus* and *Staphylococcus*) is also diet‐dependent. We hypothesize a potential role of the microbiota in MP production in *H. axyridis* as a possible example for outsourcing the synthesis of ecologically important semiochemicals to its gut bacteria.

## 1 INTRODUCTION

The volatile alkylpyrazines, particularly methyl‐ and methoxypyrazines (MPs) are heterocyclic aromatic, nitrogen‐containing compounds, which produce odors of diverse biological significance. Apart from being important constituents of flavor in food, food products, several drinks and wine, numerous pyrazines also show antibacterial, and tuberculostatic properties, making them important agents in the food processing and the biomedical research industry (Bonde, Peepliwal, & Gaikwad, 2010; Maga, 1982). Pyrazines are also widespread in nature, with diverse roles ranging from plant defense and promotion of plant growth, to serving semiochemicals in plant–insect interactions, components of insect sex pheromones, and in inter‐specific defenses within fungi (von Beeren, Schulz, Hashim, & Witte, 2011; Murray, Shipton, & Whitfield, 1970; Murray & Whitfield, 1975).

In insects, pyrazines are present in diverse orders (Moore, Brown, & Rothschild, 1990; Supporting information Table S1). Beetles, particularly in the predacious subfamily Coccinellinae (Coleoptera: Coccinellidae) containing the two‐spotted ladybeetle *Adalia bipunctata*, the seven‐pointed ladybird *Coccinella septempunctata,* the convergent ladybird *Hippodamia convergens* as well as the harlequin ladybird *Harmonia axyridis,* have evolved to use pyrazine in several inter‐ and intraspecific interactions, as allomones or pheromones, which function as deterrents or attractants (Guilford, Nicol, Rothschild, & Moore, 1987, Rizzi, 1988, Woolfson & Rothschild, 1990, Rothschild & Moore, 1987, Moore et al., 1990, Rowe & Guilford, 1999, Siddall & Marples, 2008, Verheggen, Vogel, & Vilcinskas, 2017; Supporting information Table S2). *H. axyridis* like other members of Coccinellidae releases a noxious exudate from its tibio‐femoral joints through a defensive mechanism known as reflex bleeding to deter predators. The exudate, which can account for up to 20% of their body weight, functions to release several bitter, toxic compounds having a characteristic odor, which repel vertebrate and invertebrate predators (Daloze, Brackman, & Pasteels, 1994; Hemptinne & Dixon, 2000; King & Meinwald, 1996; Majerus & Majerus, 1997; Marples, 1993; Verheggen et al., 2017). The pyrazines form an important class of compounds responsible for this characteristic repellent. Several dull colored coccinellid beetles show little pyrazine content, whereas the aposematic species show relatively higher pyrazines content (Cai, Koziel, & O'Neal, 2007; Moore et al., 1990). Thus, there could be considerable selective pressure to use pyrazines as warning signals, given the substantial energetic costs involved in their synthesis and release (Holloway, Jong, Brakefield, & Vose, 1991). Semiochemicals in Coccinellidae tracks and feces also act as warning cues leading to avoidance behavior and reduction in plant colonization by prey animals such as aphids (Ninkovic, Al Abassi, & Pettersson, 2001; Youren, 2012). The odor of coccinellid feces also has an influence on feeding and oviposition activities of conspecific as well as heterospecific competing female coccinellids (Agarwala, Yasuda, & Kajita, 2003). The three main groups of MPs found in insects are 2‐isobutyl‐3‐methoxypyrazine (IBMP), 2‐isopropyl‐3‐methoxypyrazine (IPMP), and 2‐sec‐butyl‐3‐methoxypyrazine (SBMP). They are present in diverse species of Orthoptera, Hemiptera, Lepidoptera, and Coleoptera (Moore et al., 1990; Supporting information Table S1). Besides IBMP, IPMP, and SBMP, the coccinellid beetles *H. axyridis* and *C. septempunctata* exhibit a fourth distinct MP: 2,5‐dimethyl‐3‐methoxypyrazine (DMMP) which represents a further component of the coccinellid characteristic odor (Cai et al., 2007).

Although the importance of MPs in the ecology of Coccinellinae has received considerable attention, little is known about the site and mechanism of synthesis/acquisition in these beetles, leading to the question as to how the insects acquire these multi‐purpose semiochemicals. Several plants, including *Pisum sativum*, a host plant for aphids (on which some coccinellid beetles prey) produce MPs (Murray et al., 1970). A direct correlation between MP content of plants and those of carnivorous ladybeetles is however not yet known. Ladybirds may also gather semiochemicals with additional diets such as grapes in autumn (Dunlevy et al., 2010). Additionally, MPs could also be sequestered by feeding on pyrazine‐rich prey, such as aphids (Kögel, Eben, Hoffmann, & Gross, 2012).

Besides plants, more than 350 microbial volatiles are known to be released from bacteria with pyrazines as the most prevalent substances (Dickschat, Reichenbach, Wagner‐Döbler, & Schulz, 2005, reviewed in Schulz & Dickschat, 2007). The structural similarity of some bacterial volatiles to insect pheromones suggests the possibility of microbial involvement in pyrazine synthesis in the beetles (Davis, Crippen, Hofstetter, & Tomberlin, 2013; Dickschat et al., 2005; Supporting information Table S3). Since, (a) pyrazines are a major group of volatile compounds released by bacteria, and (b) *H. axyridis* MP concentration in adults and larvae is determined by their dietary components (Kögel et al., 2012), we hypothesize that a combination of diets and gut microbiota affects MP concentrations in the beetles. In this study, we test this hypothesis by characterizing the gut microbiota of *H. axyridis* fed on different diets and its effect on the concentration of MPs.

## 2 METHODS

### 2.1 *Harmonia* rearing

The experiments were conducted with a greenhouse strain of the Asian ladybeetle *Harmonia axyridis* collected from invasive wild populations in and around Giessen (Germany) and kept in groups of about 50 individuals in cages (60 × 60 × 30 cm) according to Gegner, Schmidtberg, Vogel, & Vilcinskas, 2018. Larvae and adults were reared on the pea aphid *Acyrthosiphon pisum* on bean plants (*Vicia faber* var. minor) and maintained under a photoperiod of 16:8 hours (L:D) at average temperature of 21°C. For testing the MP concentrations under diapause‐conditions, we collected adult beetles from aggregations sites in autumn and spring found in indoor overwintering sites and kept them in cages at 4–8°C in the dark.

### 2.2 Diets

*Harmonia axyridis* are polyphagous generalist insects. They are known to feed on diverse diets including aphids (along with other insects of the suborder Sternorrhyncha), immature stages or eggs of numerous invertebrate prey, honeydew, pollen, and fruits like grapes (Berkvens et al., 2008; Botezatu, Kotseridis, Inglis, & Pickering, 2013; Galvan, Koch, & Hutchison, 2008; Koch, 2003; Roy et al., 2016). They can also be reared on eggs of the Angoumois grain moth *Sitotroga cereallella* or the flour moth *Ephesitia kuehniella* (Gegner et al., 2018; Kögel et al., 2012; Laugier et al., 2013). To investigate the effect of diet on MP synthesis, we fed the beetles with five different plant‐based and insect‐based diets. We divided the beetles into five experimental groups based on the diets they received. The beetles were offered living aphids (group I, aphid), or bisected organic grapes (group II, grape). In group III, beetles were provided with 50 µl of 4:1 honey syrup (H) diluted with water. In group IV, beetles were fed with a diet containing 500 mg eggs of *Sitotroga cerealella* mixed with 1 ml honey syrup to increase palatability (group IV, HS). In group V, the beetles were fed antibiotics through a honey syrup‐egg mass containing 1:1 tetracycline/ampicillin mix (50 µg/ml, Roth, Germany) (group V, HSAB) to study the effect of manipulation of gut microbiota on MP concentrations (modified after Hurst, Majerus, & Walker, 1992, Noriyuki, Kameda, & Osawa, 2014). Diets were kept at the periphery of Petri plates that contained moistened filter paper and housed adults (females and males) and freshly hatched first instar (L1) larvae. The ratio of females to males was retained 1:1. For adults, ad libitum food was provided daily for 10 days, while larvae were fed until they reached L4 stage or the pupal stage. At the end of the experiment, adults and larvae were dissected to remove their entire alimentary canal, and the residual body was frozen in liquid nitrogen. Another group of L4 larvae was allowed to pupate, and emerging adults were frozen 0–24 hr after hatching. The samples were stored at −20°C for gas chromatography‐mass spectrometry (GC/MS) analysis and stored in 70% ethanol at −80°C for DNA extraction.

### 2.3 Identification of gut microbiota

For analysis of the gut microbiome, we used male and female adult beetles fed on an aphid or grape diet. Beetles were washed in PBS (pH 7.2) before dissections. The gut samples were again washed in PBS and pooled in 70% ethanol. The residual bodies were also collected in 70% ethanol. This procedure was separately performed with females and males for two diets (aphids vs. grapes). Thus, we analyzed the following groups: I) guts from males fed on grapes (M‐gut‐gr), II) guts from males fed on aphids (M‐gut‐ap), III) guts from females fed on grapes (F‐gut‐gr), IV) guts from females fed on aphids (F‐gut‐ap), V) residual bodies from males fed on grapes (M‐body‐gr), VI) residual bodies from males fed on aphids (M‐body‐ap), VII) residual bodies from females fed on grapes (F‐body‐gr), and VIII) residual bodies from females fed on aphids (F‐body‐ap). DNA was extracted from pooled samples using the PowerSoil DNA extraction kit (MoBio Laboratories, USA) according to the manufacturer's recommended protocol.

To characterize the bacterial gut community, the V1–V3 region of the 16S rRNA gene was amplified from total genomic DNA using Gray28F and Gray519R primers (Sun, Wolcott, & Dowd, 2011) and sequenced using a Roche 454 FLX instrument with Titanium chemistry at an external facility (Molecular Research LP, Shallowater, TX). Sequences were quality filtered in QIIME (version 1.8.0; Caporaso, Kuczynski, et al., 2010b) using default parameters and denoised using Denoiser (Reeder & Knight, 2010). Sequences were quality filtered, chimeric sequences were removed, and high‐quality sequences were clustered using the USEARCH (Edgar, 2010) pipeline in QIIME. Taxonomy was assigned using RDP (Wang, Garrity, Tiedje, & Cole, 2007) at 80% confidence level using the with the Greengenes database version 13_8 (DeSantis et al., 2006 *)* preclustered at 97% identity. Representative sequences were aligned using PyNAST (Caporaso, Bittinger, et al., 2010a), filtered, and a phylogenetic tree was constructed using FastTree (Price, Dehal, & Arkin, 2010). For downstream analyses, the OTU table was rarefied to 15,000 sequences per sample. Data were analyzed in R (Pinheiro, Bates, DebRoy, & Sarkar, 2014) and Qiime. Heatmaps were plotted using *lattice* (Sarkar, 2010) in R with OTUs summarized at the genus level, and those classified below family level, and with cumulative relative abundance <0.1% grouped into “Others.”.

### 2.4 GC/MS analysis of MPs

For MP analysis, at least five *H. axyridis* (larvae and adults), their tissues and the diets were individually measured for each experiment. They were washed in sterile PBS before they were frozen in liquid nitrogen and stored subsequently at −20°C until analysis. Whole gut tissue along with residual body tissue was also washed and frozen. Each specimen of the different diet groups was thawed, separately weighed using an electronic balance, and transferred into a vial. Beetles and larvae were macerated with a scoop, and guts were slightly pressed with the tip of a pipette. The headspace GC/MS analsyis was modified from Cudjoe, Wiedekher, and Brindle (2005). The collected headspace samples were analyzed on a GC‐MS system (model 5977B, Agilent, Santa Clara, CA, USA) equipped with a UNIS 500 split/splitless injector (JAS, Moers, Germany). Compounds were separated on a HP‐5MS Ultra Inert GC column (Agilent) with helium as carrier gas. The MS was operated in full scan mode (EI Energy: 70 eV, transfer line: 280°C, ion source: 230°C, quadrupole: 150°C) and spectra were recorded from m/z 50‐550. Signals were integrated in extracted ion chromatograms specific for the individual MP (IPMP: m/z 137, 11.1 min; IBMP: m/z 124, 12.5 min; SBMP: m/z 138, 12.4 min; IPEP (IS): m/z 166, 12.2 min). Concentrations were calculated based on calibration curves generated using the identical IS addition and NTD sampling protocol with MP standard dilutions and normalized on a ‘per fresh weight' or ‘per sample' basis.

### 2.5 Microscopy

For light microscopy, guts were dissected while submerged in ice‐cold PBS (pH 7.2). Prefixation was performed in 2.5% glutaraldehyde plus 2% paraformaldehyde in 0.1 M cacodylate buffer (pH 7.4) for 2 hr. After washing in cacodylate buffer, the samples were post‐fixed with 1% OsO_4_ in 0.1 M cacodylate buffer (pH 7.4) for 1.5 hr, dehydrated through a graded ethanol series and embedded in Araldite. Semi‐thin sections were prepared with a Reichert Om/U3 ultramicrotome stained with 0.5% toluidine blue in 0.5% sodium borate. The samples were observed under a Leica DM 5000 B microscope.

### 2.6 Statistical analysis

Statistical analysis for the different life stages and diets was carried out using SIGMAPLOT v. 12.0 (Systat Software Inc., San Jose, CA). Significant differences between groups of parametric data were determined by one‐way analysis of variance (ANOVA) with a subsequent Holm–Sidak test. Nonparametric data were analyzed by ANOVA on ranks with a subsequent Dunn's test. For the pairwise comparison of controls and antibiotic treatments, we used IBM SPSS Statistics v23 software (Armonk, USA). Statistical differences were calculated by Mann‐Whitney U test for nonparametric data and Student's *t* test for normally distributed data. Statistical significance was defined as a threshold of *p* < 0.05 (Appendix S1: Supplementary Material Part II—Statistical Analysis and Data Set).

## 3 RESULTS AND DISCUSSION

### 3.1 MP concentrations during life cycle of *H. axyridis* reared on aphid diet

**Figure 1:**
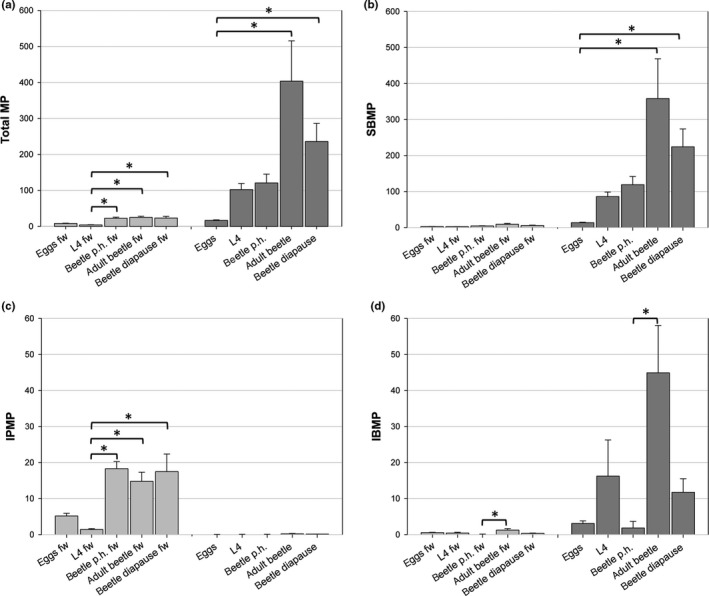
Methoxypyrazine (MP) content in life stages of the ladybird beetle *H. axyridis*. (a) Total MP content. (c) IPMP (2‐isopropyl‐3‐methoxypyrazine) content. (d) IBMP (2‐isobutyl‐3‐methoxypyrazine) content. Gray: data for MP pg/mg fresh weight (fw); dark gray: data for MP pg/sample. Life stages of *H. axyridis*: eggs, last larval instars (L4), beetles post hatching without food intake (beetle p.h.), mature beetles (adult beetle), beetles in diapause (beetle diapause). Statistical significant differences in values *p < 0.05

The total MP contents in *H. axyridis* individuals were affected by their life stages. For groups that were exclusively fed with the aphid *A. pisum*, there was an overall increase in total MP concentration in their fresh weight. Whereas larval stages showed significantly lower concentrations (mean ± *SE* = 4.192 ± 0.908 pg/mg) than adults (24.982 ± 3.356 pg/mg), MP concentrations were comparable between newly hatched adults (22.798 ± 2.645 pg/mg), mature adults, and adults in diapause (23.364 ± 4.546 pg/mg; Figure 1a; Supporting information Table S4). However, total amount of MPs (per sample) was lower in diapause‐induced adults (235.943 ± 50.244 pg/mg) than in mature adults (403.410 ± 111.993 pg/mg). Generally, the egg stage showed relatively higher MP content per mg fresh weight (8.115 ± 0.908 pg/mg) than the L4 larvae (4.192 ± 0.5 pg/mg).

The different MPs tested (IPMP, IBMP, and SBMP) varied in their relative concentrations across beetle life stages that were exclusively fed with *A. pisum* (Figure 1b; Supporting information Table S4). The most abundant of the three tested MPs was SBMP, whose overall distribution over life stages was similar to that of the total MP content. In contrast, IPMP constituted a relatively smaller proportion of the total MP fraction (Figure 1c; Supporting information Table S4). It was the lowest in L4 larvae (1.414 ± 0.231) and significantly higher in beetles (newly hatched beetle 18.286 ± 1.985 pg/mg, mature beetle 14.769 ± 2.536 pg/mg, beetle in diapause 17.486 ± 4.870 pg/mg). IBMP concentrations also constituted a relatively smaller proportion of the total MP content. However, contrary to IPMP concentrations, IBMP levels were significantly higher in mature adult beetles (pg/per mg fresh weight 1.235 ± 0.428, per individual 44.906 ± 13.137) as compared to immature beetles. In the latter group, only one individual showed a detectable IBMP level. Thus, IBMP levels appeared more important in the larval stage and in mature adult beetles (Figure 1d; Supporting information Table S4). Our result that L4 larvae of *H. axyridis* show relatively lower MP production than their adult stages corroborates with the experiments by Ninkovic et al. (2001) and Moore et al. (1990) which show lower levels of MP volatiles in larvae which can produce lower avoidance behavior in aphids.

Both non‐diapausing and diapausing beetles produced MPs (Figure 1), indicating that the potential aggregating pheromone of reflex bleeds are also produced under nonwintering conditions (Durieux et al., 2015; Jeanson & Deneubourg, 2009). However, these data also indicate the relative proportion of SBMP, IPMP, and IBMP differed between non‐diapause and diapause, such that a change in the relative proportion within the MP cocktail could enable the beetles to use the same set of volatile compounds to display different signals under different conditions (Figure 1b–d). Diapausing and non‐diapausing *A. bipunctata* produce the same three methoxypyrazines (IBMP, IBMP, and SBMP) regardless of physiological state, but IBMP (alone or combined with IPMP) can result in aggregation and causing diapause (Susset et al., 2013). Since adult beetles can secrete MPs through reflex bleeding throughout their lifetimes, including during autumnal aggregation, MPs form part of a multi‐modal display strategy in *H. axyridis*. In conjunction with other traits such as coloration, gregarious behavior, and reflex bleeding that are primarily defensive, MPs have likely been co‐opted as aggregation pheromones (Wheeler & Cardé, 2013). MPs are also associated with inducing aggregation; for example, *A. bipunctata* spend more time in the vicinity of an MP source when an extract containing cuticular hydrocarbons from diapausing individuals was present (Durieux et al., 2015; Susset et al., 2013). In *H. convergens*, IBMP has the strongest aggregative effect, whereas beetles aggregate only in specific doses of SBMP, while IPMP is repellent. IBMP is released only by adults that are on target for their overwintering locations attaining the diapause (McCord, 2015; Wheeler & Cardé, 2013). In our experiments, the adult beetles of *H. axyridis* showed the highest IBMP content (pg per mg fresh weight 1.235 ± 0.428, per individual 44.906 ± 13.137), whereas diapausing beetles had comparatively low amounts (per mg fresh weight 0.307 ± 0.102, per individual 11.728 ± 3.779) (Figure 1d; Supporting information Table S4). If IBMP also functions in inducing aggregation in *H. axyridis,* these data suggest that the release of IBMP could occur prior to beetles entering diapause, when it would function to induce aggregation among diapausing adults.

**Figure 2:**
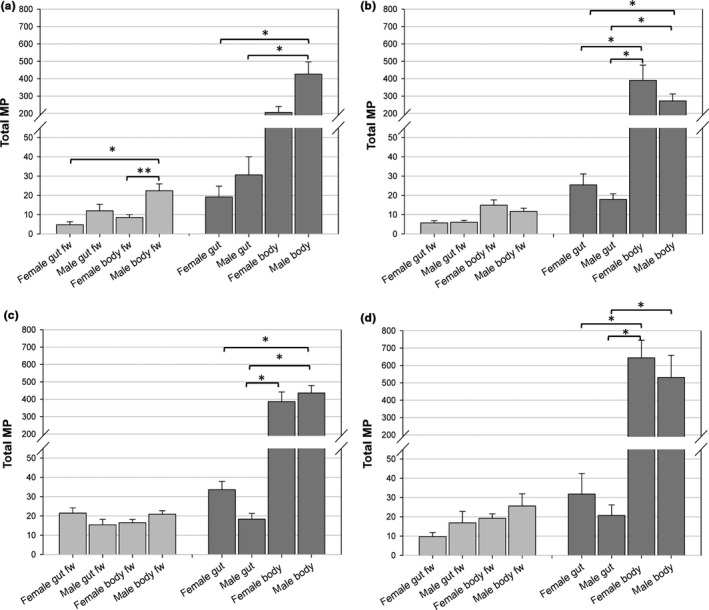
Influence of diets on total MP content in mature beetles. Quantification of MP content in gut and residual body (body) of male and female *H. axyrids*. Gray: data for MP pg/mg fresh weight (fw); dark gray: data for MP pg/sample. The following feeding assays were performed for 10 days: (a) Aphid diet on Petri dishes. (b) Grape diet. (c) Honey syrup diet. (d) Honey syrup/*S*. *cerealella* eggs diet. Statistical significant differences in values *p < 0.05; **p < 0.01.

Male *H. axyridis* beetle guts showed higher MP concentrations than females quantified in both fresh weight (males 11.956 ± 3.424 pg/mg, females 5.783 ± 1.622 pg/mg) and per individual (males 30.640 ± 9.382 pg/mg, females 20.013 ± 4.448 pg/mg) (Figure 2a; Supporting information Table S4). The magnitude of the differences was even stronger in residual body samples (males 426.478 ± 70.175 pg/mg, females 205.908 ± 34.576 pg/mg) that were analyzed after removal of the gut, indicating that beetles carry substantial MP content in extra‐intestinal tissue.

### 3.2 Source of MPs in ladybird beetles

To investigate the source of MPs in *H. axyridis*, we explored two scenarios through which the beetles could acquire or generate MPs: external acquisition through diets, or through autogenous synthesis. Potential “external” sources include sequestration of compounds from their host plants or host prey (such as aphids), or acquisition through their microbiota (von Beeren et al., 2011; Dettner, 1987; Rothschild, Euw, & Reichste, 1973; Witte, Ehmke, & Hartmann, 1990).

To test the influence of diet on the MP content of *H. axyridis*, we provided the beetles with plant‐based and insect (prey)‐based diets: *A. pisum*, grapes, honey syrup, and *S. cerealella* eggs mixed with honey (Figure 2). MPs were below the detection level and likely absent in all these diets (Appendix S1: Supplementary Material Part II—Statistical Analysis and Data Set), and thus, we ruled out these diets as a direct source of sequestering pyrazines in beetles. Despite this, all three MPs were detected in beetle life stages, and these concentrations were influenced on the diets on which they fed, the life stage of the beetles and the sex of the adults. Residual body tissue showed greater than tenfold increase in total MP content in mg/sample in all four diets. Aphid‐fed males (Figure 2a; Supporting information Table S4) showed higher MP levels than the females, significantly in the residual body (males 22.386 ± 3.552 pg/mg, females 8.514 ± 1.437 pg/mg). Generally, aphid‐fed males had higher MP concentrations in mg fresh weight (gut 11.956 ± 3.424 pg/mg, residual body 22.386 ± 3.552 pg/mg) than grape‐fed male beetles (gut 7.253 ± 1.411 pg/mg, residual body 11.717 ± 1.641 pg/mg) (Figure 2b; Supporting information Table S4). On the grape diet, the MP contents of females were generally higher than in males. Both sexes of beetles with honey syrup diet show higher MP amounts in mg/fresh weight particularly in the gut tissue (females 21.487 ± 2.692 pg/mg, males 15.384 ± 2.832 pg/mg) in comparison to the grape (females 5.741 ± 1.075 pg/mg, males 7.235 ± 1.411 pg/mg) and aphid diet (females 5.783 ± 1.622 pg/mg, males 11.956 ± 3.424 pg/mg) (Figure 2c; Supporting information Table S4). The addition of *S. cerealella* eggs to honey syrup led to the highest measured MP levels in the residual body of *H. axyridis* (pg per mg fresh weight 19.251 ± 2.259, per individual 643.689 ± 101.086; Figure 2d; Supporting information Table S4).

To further investigate the site of MP synthesis in *H. axyridis* beetles, we used different tissues from dissected beetles that were fed on an aphid diet. We isolated the beetles' hemolymph, fat body, muscle, and the alimentary canal to analyze their MP content. We found no detectable levels of total MPs in the muscle or the fat body (Appendix S1: Supplementary Material Part II—Statistical Analysis and Data Set). However, the hemolymph showed the highest concentration of MP content (37.65 ± 2.486 pg/mg fresh weight), which was approximately four times greater than MP concentration in the alimentary canal (9.07 ± 2.375 pg/mg fresh weight; Appendix S1: Supplementary Material Part II—Statistical Analysis and Data Set). As a reflex bleeder, it is likely that pyrazines are secreted from the hemolymph, facilitating a spontaneous as well as voluminous exudate during warning displays. In conclusion, these results indicated that MPs were not present in the diets themselves, but were detected in gut of the beetles that feed on these diets, with a high prevalence in their hemolymph. We therefore hypothesized that the alimentary canal is the site of synthesis of the MPs from which they are released into the hemolymph, which act as a storage tissue for release during reflex bleeding.

We thus ruled out the sequestration of MPs in *H. axyridis* either through plant sources (grapes, honey syrup) or through insect prey (aphids and *Sitotroga* eggs). An MP uptake via other food sources could also be excluded, because *H. axyridis* individuals were exclusively fed on their assigned diets throughout these experiments. Although Moore et al. (1990) examined several insect host plants for the presence of alkylpyrazines and found IPMP, IBMP, and SBMPs in Asclepiadaceae, Aristolochiaceae, Passifloraceae, Asteraceae, and Papaveraceae families, we show here that MP levels were not detected in grapes that were fed to our experimental beetles. Thus, we infer that since beetles could not acquire MPs from their diets, they must synthesize them within their body. This could be achieved either by the host themselves, or through the metabolic activity of microbial symbionts.

### 3.3 The bacteria community of the gut of *H. axyridis*

To investigate the effect of diet on the *H. axyridis* microbiome, we characterized the bacterial community of gut and residual body samples of adult beetles that were fed on either aphids or grapes. We used two approaches to characterize the effect of diet on the beetle microbiome. First, we identified a core bacterial community separately for gut tissue and residual body tissue by identifying OTUs (summarized at the genus level) that were present in 100% sample replicates for the two categories (irrespective of the diet). Secondly, we identified which OTUs were differentially abundant as an effect of the diets in the two tissue types.

**Figure 3:**
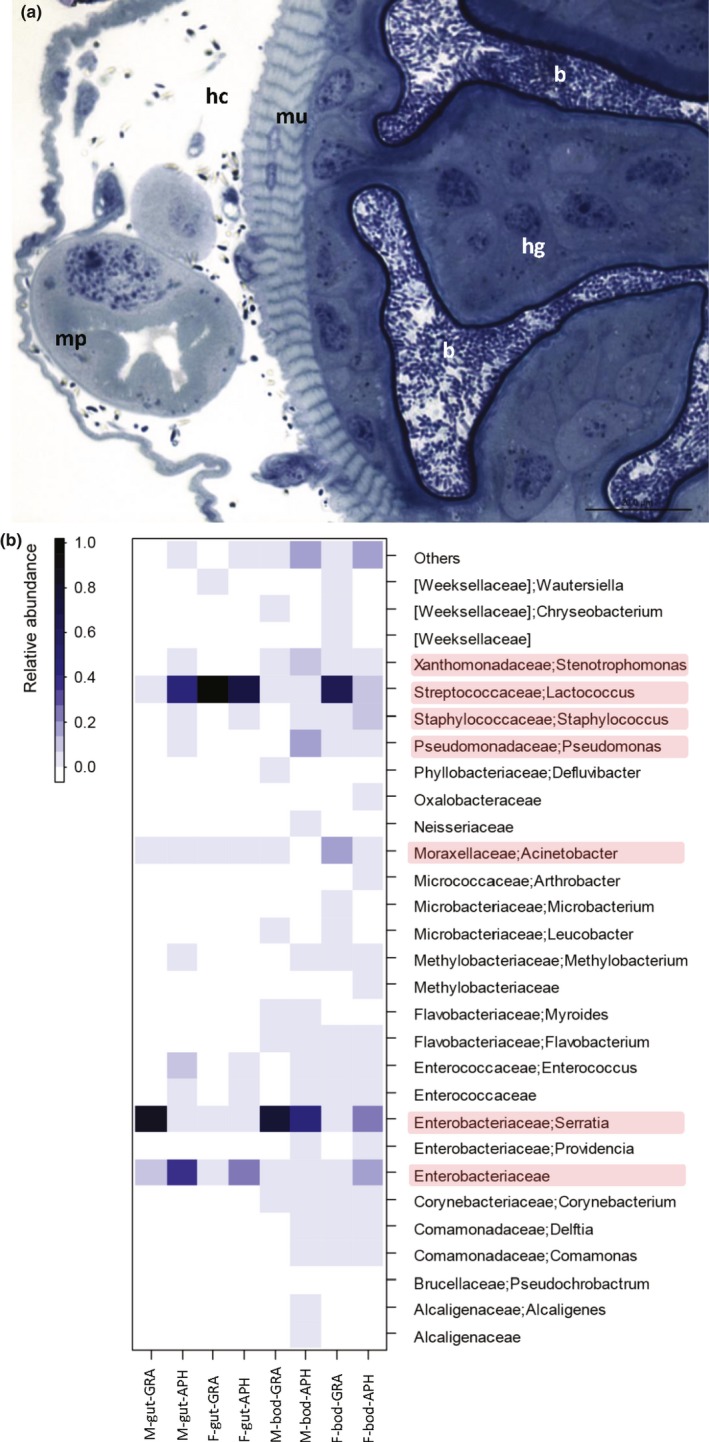
Microbiome analysis of gut of *Harmonia axyridis*. (a) Micrograph of bacteria in the lumen of the hindgut of a female *H. axyridis*. b: bacteria; hc: hemocoel, hg: hindgut; mu: muscle layer; mp: Malpighian tubules. (b) Heatmap showing relative proportion of bacteria summarized at genus level for male and female samples (gut tissue and residual body tissue) for adult beetles fed on aphid and grape diets. ap: aphid diet; bod: residual body tissue; M: males; F: females; gr: grape diet. Bacteria highlighted in red are presumed to produce MPs

A total of 14 OTUs were identified as the core bacterial community for the male and female gut tissue. These included the genera *Lactococcus*,* Serratia*, and unclassified Enterobacteriaceae and Enterococcaceae. The core community for the residual body tissue for adults consisted of 35 OTUs consisting *Lactococcus*,* Staphylococcus*,* Serratia*,* Corynebacterium*,* Delftia*,* Pseudomonas*,* Stenotrophomonas*,* Acinetobacter*,* Providencia*,* Methylobacterium*,* Flavobacterium*,* Comamonas*, and several unclassified families. Proteobacteria and Firmicutes were the two most dominant bacterial phyla in male and female samples (Figure 3). At the species level, some *Lactococcus* OTUs were assigned to *L. garvieae*, while the most abundant OTU was identified as *L. lactis* (BLAST against the nr database, 98% sequence identity). The *Serratia* OTUs were identified as *S. marcescens* (BLAST against the nr database, 98% sequence identity), and the Enterobacteriaceae were identified as *Enterobacter hormaechei* (BLAST against the nr database, 98% sequence identity; Table 1).

**Table 1:** Bacterial community of the gut of *H. axyridis*

Bacteria in gut of *H. axyridis*				MP production verified in strains or closely related strains
Phylum	Order	Family	Genus/species	
Firmicutes	*Bacillales*	Staphylococcaceae	*Staphylococcus kloosii*	***Staphylococcus sciuri*** *, S. aureus* (Robacker, 2007; Robacker et al., 1991, 1998)
			*Staphylococcus succinus*	
			***Staphylococcus sciuri***	
	*Lactobacillales*	Enterococcaceae	Unclassified Enterococcaceae	No information
			*Enterococcus*	
			*Enterococcus mundtii*	
		Streptococcaceae	*Lactococcus*	***Lactococcus lactis* subsp. *lactis*** biovar. *diacetilactis* (Heon & Lee, 1991; Lee, DeMilo, Moreno, & Martinez, 1995)
			***Lactococcus lactis *subsp.*lactis***	
			*Lactococcus garvieae*	
	Bacillales			See Table 2
γ‐Proteobacteria	Enterobacteriales	Enterobacteriaceae	Unclassified Enterobacteriaceae	*Enterobacter agglomerans* (Robacker et al., 1998, Robacker & Lauzon, 2002, Robacker et al. 2007)
			*Enterobacter hormaechei*	
			*Serratia * sp.	***Serratia marcescens*** *, S. ficaria, S. rubidea, S. odorifera* (Gallois & Grimont, 1985)
			***Serratiamarcescens***	
	Aeromonodales	Aeromonadaceae	*Aeromonas* sp.	no information
	Pseudomonaldales	Pseudomonadaceae	*Pseudomonas*	*Pseudomonas perolens, P. taetroleus, P. aeruginosa* (Morgan et al., 1972, Miller et al., 1973, Dumont et al., 1983, Gallois et al., 1988, Cheng et al., 1991, Cheng & Reineccius 1991)
		Moxarellaceae	*Acinetobacter*	*Acinetobacter baumannii* (Gao et al., 2016)
	Xanthomonadales	Xanthomonadaceae	*Stenotrophomonas*	*Stenotrophomonas maltophilia* (Zou et al., 2007)
α‐Proteobacteria	Rhizobiales	Methylobacteriaceae	*Methylobacterium*	*Methylobacterium* sp.: diverse pyrazines (Balachandran, Duraipandiyan, & Ignacimuthu, 2012)
Bacteroidetes	Flavobacteriales	Flavobacteriaceae	*Wautersiella*	no information

Note

Bold: species with already described MP production and the respective references; underlined: core bacteria of the gut of *H. axyridis*.

Several OTUs showed differential relative abundances based on the tissue sampled and the diets. Although, OTUs assigned to the genus *Lactococcus* were present in all samples, they showed higher relative abundances in female and male gut tissue with aphid diets and female gut tissue with grape diet. *Serratia* showed highest relative abundance in male body and male gut tissue fed on grapes. *Corynebacterium* was exclusively found in residual body tissue in males and females. Based on the Bray‐Curtis dissimilarity measure, the female gut tissues across diets clustered together, indicating greater similarity in gut microbiomes in females despite feeding differences.

The bacteria community composition described here shows overlap with the community composition of native *H. axyridis* (from Korea), which consisted of α‐ and γ‐*Proteobacteria*,* Actinobacteria*,* Firmicutes,* and *Deinococcus–Thermus* (Kim, Han, Moon, Yu, & Whang, 2011; Moon et al., 2011). In contrast, the beetles used in this study showed *Enterococcus* and *Lactococcus* strains which were not identified in samples of native *H. axyridis*. However, the microbiome of invasive *H. axyridis* from Poland encompassed Lactococcus, but not Enterococcus (Dudek, Humińska, Wojciechowicz, & Tryjanowski, 2017). However, it remains uncertain whether the differences in these taxa were an effect of diet.

Some of the bacteria we detected in the gut of *H. axyridis* are known producers of methoxypyrazines (Table 1): viz. Firmicutes with the genus *Lactococcus,* and particularly the species *Lactococcus lactis* subsp*. lactis* and the species S*taphylococcus sciuri*; γ‐Proteobacteria with the genera *Serratia*,* Pseudomonas*,* Acinetobacter*,* Stenotrophomonas*, and those belonging to the family Enterobacteriaceae. For example, *Lactococcus lactis* subsp*. lactis*, which was highly abundant in male and female beetles in this study, produces tetramethylpyrazines (Heon & Lee, 1991). Several *Serratia* species are also known to generate MPs (Dalamaga & Vrioni, 2011; Gallois & Grimont, 1985; Tables 1 and 2). In the current study, *Serratia* was relatively more abundant in males. It is presumed that *Serratia* strains in the insect digestive tract probably originate from plants (Grimont & Grimont, 2006), and it is known that several *Serratia* strains generate potato‐like odors with a combination of different pyrazine compounds such as 3‐isopropyl‐2‐methoxy‐5‐methylpyrazine, 3‐sec‐butyl‐2‐methoxy‐5(6)‐methylpyrazine, and 3‐isobutyl‐2‐methoxy‐6‐methylpyrazine (Gallois & Grimont, 1985) (Table 2). *Staphylococcus* was detected in male and female tissue of beetles feeding on aphids and is also known to produce methoxypyrazines (Robacker, 2007; Robacker, DeMilo, & Voaden, 1991; Robacker, Martinez, Garcia, & Bartelt, 1998). In aphids, *S. sciuri* is known to produce volatile semiochemicals that attract natural enemies. Bacteria of the genus *Pseudomonas* are known to produce IPMP and SBMP (Chen & Reineccius, 1991; Cheng, Reineccius, Bjorklund, & Leete, 1991; Dumont, Mourgues, & Adda, 1983; Filipiak et al., 2012; Gallois, Kergomard, & Adda, 1988; Miller, Scanlan, Lee, Libbey, & Morgan, 1973; Morgan, Libbey, & Scanlan, 1972) and Kim et al. (2011) and Moon et al. (2011) also described two *Pseudomonas* strains in native *H. axyridis*. Bacteria associated with *Acinetobacter* and *Stenotrophomonas* that were detected in the gut tissue of adults feeding on aphids are reported to synthesize pyrazines (Gao et al., 2016; Zou, Mo, Gu, Zhou, & Zhang, 2007).

**Table 2:** “*Harmonia”* MPs and others produced by bacteria and fungi

Origin—bacteria/fungi	Mainly produced pyrazines	Reference
*Enterobacter agglomerans* from mouthparts of* Anastrepha ludens* and *Rhagoletis pomonella*	2,5‐dimethylpyrazine, trimethylpyrazine	Robacker et al. (1998), Robacker, Lauzon, and He (2004), Robacker and Lauzon (2002)
*Cedecea davisae*	SBMP, 3‐isopropyl‐2‐methoxypyrazine, IBMP	Dalamaga and Vrioni (2011), Gallois and Grimont (1985)
*Serratia ficaria*	IPMP, 3‐s‐butyl‐2‐methoxy‐5(6)‐methylpyrazine	
*S. marcescens*	3‐isopropyl‐2‐methoxy‐5‐methylpyrazine, 2,3,5‐trimethylpyrazine	
*S. odorifera*	3‐isopropyl‐2‐methoxy‐5‐methylpyrazine, 3‐isobutyl‐2‐methoxy‐6‐methylpyrazine	
*S. rubidaea*	3‐isopropyl‐2‐methoxy‐5‐methylpyrazine, 3‐s‐butyl‐2‐methoxy‐5(6)‐methylpyrazine, 2,3,5‐trimethylpyrazine, 2‐ethyl‐6‐methylpyrazine	
*Citrobacter freundii*	2,5‐dimethylpyrazine	DeMilo, Lee, Moreno, and Martinet (1996), Robacker and Bartelt (1997), Robacker (2007)
*Klebsiella pneumoniae*	2,5‐dimethylpyrazine, 2‐isopropyl‐5‐methylpyrazine	Martinez, Robacker, Garcia, and Esau (1994), Lee et al. (1995), Robacker et al. (2004), Robacker (2007), Schulz and Dickschat (2007)
*Pseudomonas perolens*	IPMP, SBMP	Morgan et al. (1972), Miller et al. (1973), Dumont et al. (1983), Cheng et al. (1991), Cheng & Reineccius (1991)
*Pseudomonas taetrolens*	IPMP	Gallois et al. (1988)
*Acinetobacter baumannii*	2,5‐dimethylpyrazine	Gao et al. (2016)
*Stenotrophomonas maltophilia*	2,3,5‐trimethylpyrazine, 2,5‐dimethylpyrazine	Zou et al. (2007)
*Staphylococcus aureus* isolated from *A. ludens*	2,5‐ dimethylpyrazine	Rohbacker and Flath (1995), Robacker et al. (1998), Robacker (2007)
*Lactococcus lactis* subsp *lactis* biovar. *diacetilactis*	tetramethylpyrazine	Heon and Lee (1991), Lee et al. ( 1995)
*Bacillus subtilis*	2,5‐dimethylpyrazine, 2,3,5,6‐tetramethylpyrazine	Besson, Creuly, Gros, and Larroche (1997), Larroche, Besson, and Gros (1999), Xiao, Xie, Liu, Hua, and Xu (2006), Zou et al. (2007), Zhu, Xu, and Fan (2009)
*Bacillus licheniformes*	2,3 dimethylpyrazine, trimethylpyrazine, tetramethylpyrazine	Zhang, Wu, and Xu (2013)
*Bacillus thuringiensis*	2,5‐ dimethylpyrazine	Robacker et al. (1991), Robacker et al. (1998), Robacker (2007)
*Bacillus sphaericus*	2,5‐ dimethylpyrazine	
*Bacillus megaterium*	2,5‐ dimethylpyrazine	
*Bacillus popilliae*	2,5‐ dimethylpyrazine	
*Paenibacillus polymyxa*	tetramethylpyrazine, methylethylpyrazine, 2,5‐di(propan‐2‐yl)pyrazine, 2,5diisopropylpyrazine	Beck, Hansen, and Lauritsen (2003), Schulz and Dickschat (2007)
*Alicyclobacillus acidoterrestris* isolated from apple juice	IBMP	Siegmund and Pöllinger‐Zierler (2006)
*Micrococcus luteus*	2,5‐ dimethylpyrazine	Robacker et al. (1991), Robacker et al. (1998), Robacker (2007)
*Actinomycetes* sp isolated from apple juice	IBMP	Siegmund and Pöllinger‐Zierler (2006)
*Corynebacterium glutamicum*	tetramethylpyrazine	Dickschat et al. (2010)
*Chondromyces crocatus*	2,5‐dialkylpyrazine, 3‐methoxy‐2,5‐dialkylpyrazine (side‐chains: methyl, isopropyl, isobutyl, or sec‐butyl)	Dickschat et al. (2005)
unclassified on fruit surface	2,5‐diisopropylpyrazine	Zilkowski, Bartelt, Blumberg, James, and Weaver (1999)
unclassified	3,5‐dimethyl‐2‐methoxypyrazine	Mottram and Patterson (1984)
unclassified	2,5‐diisopropylpyrazine	Zilkowski et al. (1999)
*Aspergillus parasiticus*	IPMP, 2‐hydroxy‐3,6‐di‐sec‐butylpyrazine	Buchanan and Houston (1982)
*Septoria nodorum*	IPMP	Devys, Bousquet, Kollmann, and Barbier (1978)

Note

IBMP: 2‐isobutyl‐3‐methoxypyrazine; IPMP: 2‐isopropyl‐3‐methoxypyrazine); SBMP: 2‐sec‐butyl‐3‐methoxypyrazine.

### 3.4 Role of gut bacteria on the MP production

**Figure 4:**
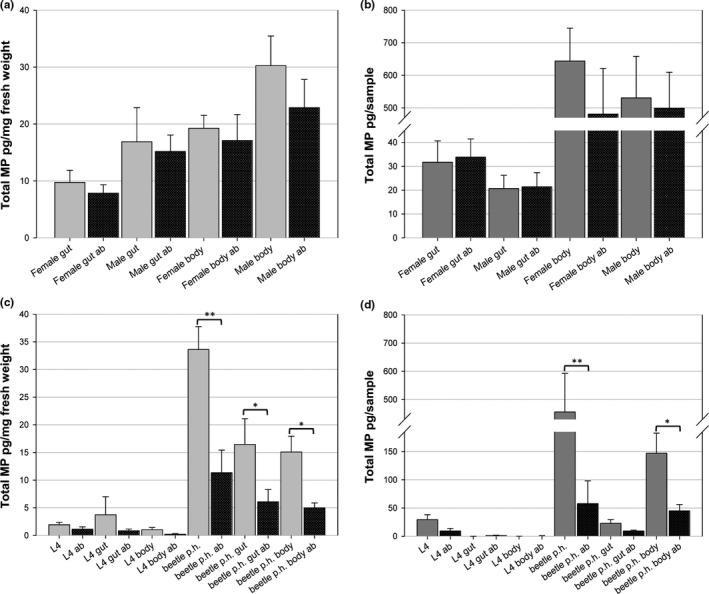
Influence of antibiotics on total MP content of *H. axyridis*. A Total MP concentration (pg/mg) fresh weight of guts and residual bodies (body) of female and male *H. axyridis* fed for 10 days with *Sitotroga* eggs (in honey) (gray) and *Sitotroga* eggs mixed with honey and antibiotics (dark spotted). (b) Total MP contents in pg/sample after same procedure as shown in 4A. (c) Total MP (pg/mg) fresh weight of whole individuals, dissected guts (gut) and residual bodies (body) from L4 larval instars and newly hatched beetles (beetle p.h.) under *Sitotroga* eggs (gray) and *Sitotroga* eggs‐antibiotic mix (dark spotted). (d) Total MP pg/sample of experiment presented in C. Statistical significant differences in values *p < 0.05; **p < 0.01

To investigate the role of gut bacteria in MP production in *H. axyridis*, we fed adult beetles with *S. cerealella* eggs (containing honey) or a HS diet supplemented with ampicillin and tetracycline (HSAB) for 10 days (Figure 4). The HSAB‐fed beetles were then dissected, and the total MP content was analyzed in their gut tissue and residual body tissue. Antibiotic‐fed beetles showed lower MP concentrations (per mg fresh weight) compared to the control beetles, both in residual body tissues as well as in the gut tissues (Figure 4a; Supporting information Table S4). There was reduction in MP levels in the residual body tissue of antibiotic‐fed males and females, but no difference in the gut tissues when MP concentrations were analyzed per sample (Figure 4b; Supporting information Table S4).

Diets can have considerable impact on MP levels at larval stages (Kögel et al., 2012); hence, we analyzed MP concentrations in larval stages and newly eclosed beetles fed on the HSAB diet. HSAB‐fed larvae had lower MP concentration than the controls, both in the gut as well as the residual body tissue. A stronger trend was also observed in newly hatched beetles, where HSAB‐fed beetles showed significantly reduced MP concentrations (control beetles 33.622 ± 4.134 pg/mg, HSAB‐fed beetles 11.329 ± 4.099 pg/mg), also in the gut (control 16.438 ± 4.674 pg/mg, HSAB 6.077 ± 2.219 pg/mg) and in the residual body tissue (control 15.092 ± 2.842 pg/mg, HSAB 4.997 ± 0.872 pg/mg) (Figure 4c; Supporting information Table S4). Thus, these data indicate that the gut microbiota seems important for MP synthesis, especially in early life stages of the beetles. We further show, that dysbiosis (the imbalance of the microbial gut community) at larval stages, can influence MP levels at adult stages, indicating that larval gut community could have lifelong influences on the beetles physiology and display signaling, if the native gut microbiota is disrupted.

## 4 CONCLUSIONS

The secretion of repellents containing methoxypyrazines through reflex bleeds is an important ecological adaptation in several insects, especially in the family Coccinellidae. The invasive ladybird beetle *H. axyridis* is known to coadapt multiple pyrazines in its reflex bleeds, which function in defense, aggregation and as warning signals. In this study, we quantified MPs in *H. axyridis* and found that MP concentrations vary across beetle life stages, sexes, and physiological status. We rule out their plant‐based and insect (prey)‐based diets as a potential source of these pyrazines, but show several fold higher accumulation in adult stages, indicating that beetles generate pyrazines endogenously or through their microbiota. Further, adults showed relatively high MP concentrations in the gut and the hemolymph, but not in the fat body or other tissue. Manipulating the gut microbiota of larvae with antibiotics significantly reduced MP concentrations. Thus, we hypothesize that the gut is the site for MP synthesis possibly involving the gut microbiota, from where it is transported to the hemolymph for release through reflex bleeds. The abundance of bacteria particularly of the genera *Lactobacillus* and *Serratia* which are known MP producers supports this hypothesis.

## CONFLICT OF INTEREST

None declared.

## AUTHOR CONTRIBUTIONS

HS performed developmental and diet‐dependent studies, microscopic analysis, data analysis, and wrote the first draft of the paper. RH designed, organized, and performed the GC/MS analysis. SPS designed, performed and described the microbiome analysis. AV and HV conceived and designed the work, and revised and edited the manuscript.

## Data Availability

Microbiome 16S amplicon sequences and data descriptions, methoxypyrazine GC‐MS data and supplemental figures are deposited in the Open Access Data Repository EDMOND and can be directly accessed at the following https://doi.org/10.17617/3.1c.
