# Supplementary material for: Symbiont‐mediated chemical defense in the invasive ladybird Harmonia axyridis
Source: Ecol Evol. 2019 Jan 25;9(4):1715–29. doi: 10.1002/ece3.4840 (PMC6392489; doi:10.1002/ece3.4840)
Supplement: Supplementary file 1 [file ECE3-9-1715-s001.pdf]

Supplementary Material Part I - Supplementary Tables

Supplementary Table 1. "*Harmonia*"-MPs in insects and their function (according to El-Sayed 2016). Bold: presumed function as pheromones in few insect species. 3-sec-butyl-2-methoxypyrazine (SBMP), 3-isopropyl-2-methoxy-pyrazine (IPMP), 3-isobutyl-2-methoxypyrazine (IBMP), 3,5-dimethyl-2-methoxy-pyrazine (DMMP).

order	species	MPs	function	reference
**Lepidoptera**	*Actinote pelleria*	IBMP	allomone	Moore et al. 1990
	Amata sp	SBMP, IPMP	allomones	Rothschild et al. 1984
	Arctica caja	IBMP, SBMP	allomones	Moore et al. 1990
	Athrophaneura aristolochiae	IBMP, SBMP	allomones	
	Athrophaneura kotzebua	IBMP, SBMP	allomones	
	Battus polydamas	IBMP, SBMP	allomones	
	Danaus plexippus	IBMP, SBMP, IPMP	allomones	
	Dryas iulia	IBMP, SBMP, IPMP	allomones	
	Euplagia quadripunctata	IBMP, SBMP	allomones	
	Heliconius atthis	IBMP, SBMP	allomones	
	Heliconius charitonia	IBMP, SBMP, IPMP	allomones	
	Heliconius melpomene	IBMP, SBMP, IPMP	allomones	
	Papilio rumanzovia	IBMP, SBMP	allomones	
	Pollanisus sp	SBMP	allomone	
	Tyria jacobaeae	SBMP	allomone	
	Zerynthia polyxena	IBMP	allomone	
	Zygaena lonicerae	IBMP, SBMP	allomones	Rothschild et al. 1984
**Coleoptera**	*Adalia bipunctata*	IBMP, IPMP	**pheromones**	Susset et al. 2013
	Calopteron reticulatum	IPMP	allomone	Eisner et al. 2008
	Calopteron terminale	IPMP	allomone	
	Coccinella septempunctata	IPMP (in ladybeetle treated wines also: SBMP, IBMP, DMMP)	**pheromone**	Petterson et al. 1999, Cudjoe et al. 2005, Botezatu & Pickering 2012
	Coccinella transversalis	SBMP, IPMP	allomones	Moore et al. 1990
	Epilachna curcurbitae	IPMP	allomone	
	E. vigintisexpunctata	IBMP, SBMP, IPMP	allomones	
	Eumorphus tetraspilotus	SBMP	allomone	
	Harmonia axyridis	IBMP, SBMP, IPMP, DMMP	**pheromones**	Pickering et al. 2004, 2005, 2008, Cudjoe et al. 2005, Cai et al. 2007
	Harmonia conformis	SBMP	allomone	Moore et al. 1990
	Hippodamia convergens	IBMP, SBMP, IPMP	**pheromones**	Cudjoe et al. 2005, Wheeler & Cardé 2013
	Illeis sp	SBMP	allomone	Moore et al. 1990
	Metriorrhynchus rhipidus	SBMP	allomone	
	Micraspis frentanta	SBMP	allomone	
	Palaestra foveicollis	SBMP	allomone	
	Pseudolycus haemopterus	SBMP	allomone	
	Rhagonycha fulva	SBMP	allomone	
	Rodatus boucardi	IPMP	allomone	
	Zonitis lutea	IBMP, SBMP, IPMP	allomones	
	Lyzus sp	IPMP	allomone	Eisner et al. 2008
**Hemiptera**	*Cercopis vulnerata*	SBMP	allomone	Körner 2006
	Murgantia histrionica	SBMP, IPMP	**pheromones**	Aldrich et al 1996
	Oncopeltus fasciatus	IBMP	allomone	
**Orthoptera**	*Poekilocerus bufonius*	SBMP	allomone	Moore et al. 1990

Supplementary Table 2. Described functions of MPs as pheromones (sex, communication, and aggregation) and/or allomones in coccinellid beetles. SBMP: 3-sec-butyl-2-methoxypyrazine, IPMP: 3-isopropyl-2-methoxy-pyrazine), IBMP: 3-isobutyl-2-methoxypyrazine.

species	pheromone: attraction (sex) and communication	aggregation pheromone	defense substance	reference
Adalia bipunctata		IBMP, IPMP	adaline, adalinine	Susset et al. 2013, Lognay et al. 1996, Tursch et al. 1973, 1975
Coccinella septempunctata	IPMP		coccinelline, precoccinelline	Tursch et al. 1975, Al Abassi et al. 1998, Cudjoe et al. 2005
Harmonia axyridis	IBMP, IPMP, SBMP	beta-caryophyllene	harmonine	Alam et al. 2002, Cudjoe et al. 2005, Verheggen et al. 2007
Hippodamia convergens	IBMP, IPMP, SBMP	IBMP	harmonine, hippodamine, convergine	Braconnier et al. 1985, Tursch et al. 1974, Cudjoe et al. 2005, Wheeler et al. 2013

Supplementary Table 3. Microbial pyrazines and the responding insects.

bacterial species	pyrazine	responding insect	reference
Klebsiella pneumoniae	2,5-dimethylpyrazine<br>trimethylpyrazine	*Anastrepha ludens*	Martinez et al. 1994, Lee et al. 1995, Rohbacker and Bartelt 1997, Robacker et al. 2004, Rohbacker 2007
Citrobacter freundii	2,5-dimethylpyrazine<br>trimethylpyrazine	*Anastrepha ludens*	DeMilo et al. 1996, Rohbacker and Bartelt 1997, Robacker et al. 2004, Robacker 2007
Enterobacter agglomerans isolated from mouthparts from *Anastrepha ludens* and *Rhagoletis pomonella*	2,5-dimethylpyrazine<br>trimethylpyrazine	*Anastrepha ludens* , *A. suspense*, *Rhagoletis mendax*, *R. pomonella*, *Schistocerca gregaria*	Lauzon et al. 1998, Robacker et al. 1998, Robacker und Lauzon 2002, Robacker et al. 2004, McCollum et al. 2009
unclassified bacteria on fruit surfaces	2,5-diisopropylpyrazine	*Carpophilus humeralis*	Zilowski et al. 1999
Paenibacillus polymyxa	tetramethylpyrazine, methylethylpyrazine, 2,5-di(propan-2-yl)pyrazine, 2,5-diisopropylpyrazine	*Carpophilus humeralis*	Beck et al. 2003, Schulz and Dickschat 2007
Staphylococcus aureus	2,5-dimethylpyrazine	*Anastrepha ludens*	Robacker and Moreno 1995
Staphylococcus sciuri	2,5-dimethylpyrazine	*Episyrphus balteatus*	Leroy et al. 2011

Supplementary Table 4. GC/MS-analysis. Mean values ± SE of MP contents (pg/mg fresh weight and pg/sample) in all performed feeding experiments. n = number of tested individuals/tissues. H = honey syrup diet, HS = honey syrup-*Sitotroga* egg diet, HSAB = honey syrup-*Sitotroga* egg-antibiotic mix diet

Figure	MP	diet	sample	n	mean pg/mg fw	SE pg/mg fw	mean pg/sample	SE pg/sample
Fig. 1A	total MP	aphid	egg	5	8.115	± 0.908	16.648	± 1.,574
			L4	6	4.192	± 0.571	102.403	± 16.753
			beetle p.h.	5	22.798	± 2.645	120.904	± 24.274
			adult	5	24.982	± 3.356	403.410	± 111.993
			beetle diapause	5	23.364	± 4.546	235.943	± 50.244
Fig. 1B	SBMP	aphid	egg	5	2.418	± 0.356	13.521	± 1.273
			L4	6	2.360	± 0.307	86.106	± 12.307
			beetle p.h.	5	4.446	± 0.730	118.997	± 22.595
			adult	5	8.978	± 2.509	358.312	± 110.153
			beetle diapause	5	5.570	± 1.300	224.087	± 49.627
Fig. 1C	IPMP	aphid	egg	5	5.163	± 0.754	0.052	± 0.006
			L4	6	1.414	± 0.231	0.079	± 0.018
			beetle p.h.	5	18.286	± 1.985	0.084	± 0.012
			adult	5	14.769	± 2.536	0.192	± 0.106
			beetle diapause	5	17.486	± 4.870	0.128	± 0.026

Fig. 1D	IBMP	aphid	egg	5	0.534	± 0.114	3.074	± 0.723
			L4	6	0.419	± 0.231	16.218	± 10.044
			beetle p.h.	5	0.066	± 0.066	1.823	± 1.823
			adult	5	1.235	± 0.428	44.906	± 13.137
			beetle diapause	5	0.307	± 0.102	11.728	± 3.779
Fig. 2A	total MP	aphid	female gut	5	5.783	± 1.622	20.013	± 4.448
			female residual body	10	8,514	± 1.437	205.908	± 34.576
			male gut	6	11.956	± 3.424	30.640	± 9.382
			male residual body	8	22.386	± 3.552	426.478	± 70.175
Fig. 2B	total MP	grape	female gut	5	5.741	± 1.075	25.414	± 5.698
			female residual body	9	14.898	± 2.775	390.081	± 87.958
			male gut	6	7.253	± 1.411	17.888	± 2.977
			male residual body	12	11.717	± 1.641	272.109	± 40.422
Fig. 2C	total MP	H	female gut	8	21.487	± 2.692	33.604	± 4.275
			female residual body	7	16.476	± 1.695	386.058	± 55.955
			male gut	6	15.384	2.832	18.312	± 3.002
			male residual body	6	20.897	± 1.791	436.080	± 42.533
Fig. 2D	total MP	HS	female gut	7	9.735	± 2.139	31.678	± 8.965
			female residual body	7	19.251	± 2.259	643.689	± 101.086
			male gut	6	16.887	± 5.985	20.675	± 5.529
			male residual body	6	25.630	± 6.286	530.780	± 127.378
Fig. 4A, B	total MP	HS	female gut	7	9.735	± 2.139	31.678	± 8.965
			male gut	7	16.887	± 5.985	20.675	± 5.529
			female residual body	6	19.251	± 2.259	643.689	± 101.086
			male residual body	6	25.630	± 6.286	530.780	± 127.378
		HSAB	female gut ab	8	7.855	± 1.486	33.802	± 7.646
			male gut ab	8	15.181	± 2.891	21.364	± 5.961
			female residual body ab	8	17.093	± 4.576	480.853	± 140.491
			male residual body ab	7	22.879	4.957	499.091	± 110.338
Fig. 4C, D	total MP	HS	L4	5	1.928	± 0.434	29.421	± 8.745
			L4 gut	5	3.733	± 3.224	0.031	± 0.019
			L4 residual body	5	1.029	± 0.377	0.114	± 0.035
			beetle p.h.	8	33.622	± 4.134	456.172	± 136.497
			beetle p.h. gut	6	16.438	± 4.674	22.916	± 6.478
			beetle p.h. residual body	6	15.092	± 2.842	147.059	± 35.642
		HSAB	L4 ab	5	1.121	± 0.416	9.222	± 4.649
			L4 gut ab	5	0.842	± 0.314	1.084	± 0.685
			L4 residual body ab	5	0.225	± 0.101	0.538	± 0.494
			beetle p.h. ab	5	11.329	± 4.099	57.882	± 40.270
			beetle p.h. gut ab	5	6.077	± 2.219	9.193	± 1.672
			beetle p.h. residual body ab	5	4.997	± 0.872	44.926	± 11.221

References for Supplementary Tables

Al Abassi, S., Birkett, M.A., Pettersson, J., Picket, J.A., & Woodcock, C.M. (1998). Ladybird beetle odor identified and found to be responsible for attraction between adults. Cellular and Molecular Life Sciences 58(8), 876-879.

Alam, N., Choi, I.S., Song, K.-S., Hong, J., Lee, C.O., & Jung, J.H. (2002). A new alkaloid from two coccinellid beetles *Harmonia axyridis* and *Aiolocaria hexaspilota*. Bulletin of the Korean Chemical Society 23, 497-499.

Aldrich, J.R., Avery, J.W., Lee, C.-J., Graf, J.C., Harrison, D.J., & Bin, F. (1996). Semiochemistry of cabbage bugs (Heteroptera: Pentatomidae: Eurydema and Murgantia). Journal of Entomological Science 31, 172-182.

Beck, H.B., Hansen, A.M., & Lauritsen, F.R. (2003). Novel pyrazine metabolites found in polymyxin biosynthesis by *Paenibacillus polymyxa*. FEMS Microbiology Letters 220, 67-73.

Botezatu, A., & Pickering, G.J. (2012). Determination of ortho- and retronasal detection thresholds and odor impact of 2,5-dimethyl-3-methoxypyrazine in wine. Journal of the Science of Food and Agriculture 77 (11), 394-398.

Braconnier, M.F., Braekman, J.C., Daloze, D., & Pasteels, J.M. (1985). (Z)-1, 17-diaminooctadec-9-ene, a novel aliphatic diamine from Coccinellidae. Experientia 41, 519-520.

Cai, L.S., Koziel, J.A., & O'Neal, M.E. (2007). Determination of characteristic odorants from *Harmonia axyridis* beetles using in vivo solid-phase microextraction and multidimensional gas chromatography-mass spectrometry-olfactometry. Journal of Chromatography A 1147, 66-78.

Cudjoe, E., Wiederkehr, T.B., & Brindle, I.D. (2005). Headspace gas chromatography-mass spectrometry: a fast approach to the identification and determination of 2-alkyl-3-methoxypyrazine pheromones in ladybugs. Analyst 130, 152-155.

El-Sayed, A.M. (2016). The Pherobase: Database of pheromones and semiochemicals. http://www.pherobase.com

Eisner, T., Schroeder, F.C., Snyder, N., Grant, J.B., Aneshansley, D.J., Utterback, D., Meinwald, J., & Eisner, M. (2008). Defensive chemistry of lycid beetles and of mimetic cerambycid beetles that feed on them. Chemoecology 18, 109-119.

Khrimian, A., Shirali, S., Vermillion, K.E., Siegler, M.A., Guzman, F., Chauhan, K., Aldrich, J.R., & Weber, D.C. (2014). Determination of the stereochemistry of the aggregation pheromone of harlequin bug, *Murgantia histrinoica*. Journal of Chemical Ecolology 40, 1260-1268.

Körner, M. (2006). Zur Rolle der Hämolymph-Inhaltsstoffe bei der Feindabwehr von Zikaden (Cicadomorpha et Fulgoromorpha) unter besonderer Berücksichtigung der Blutzikade *Cercopis vulnerata* Rossi. PhD thesis, University of Bayreuth, Germany.

Lauzon, C.R., Sjogren, R.E., Wright, S.E., & Prokopy, R.J. (1998). Attraction of *Rhagoletis pomonella* (Diptera: Tephritidae) flies to odor of bacteria: apparent confinement to specialized members of Enterobacteriaceae. Environmental Entomology 27, 853-857.

Lee, C.J., DeMilo, A.B., Moreno, D.S., & Martinez, A.J. (1995), Analyses of the volatile components of a bacterial fermentation that is attractive to the Mexican fruit fly, *Anastrepha ludens*. Journal of Agricultural and Food Chemistry 43, 1348-1351.

Leroy, P.D., Sabri, A., Heuskin, S., Thonart, P., Lognay, G., Verheggen, F.J., Francis, F., Brostaux, Y., Felton, G.W., & Haubruge, E. (2011). Microorganisms from aphid honeydew attract and enhance the efficiency of natural enemies. Nature Communications. doi: 10.1038/ncomms1347

Lognay, G., Hemptinne, J.L., Chan, F.Y., Gaspar, C.H., Marlier, M., Braekman, J.C., Daloze, D., & Pasteels, J.M. (1996). Adalinine, a new piperidine alkaloid from the ladybird beetles *Adalia bipunctata* and *Adalia decempunctata*. Journal of Natural Products. 59, 510-511.

Martinez, A.J., Robacker, D.C., Garcia, J.A., & Esau, K.L. (1994). Laboratory and field olfactory attraction of the Mexican fruit fly (Diptera: Tephritidae) to metabolites of bacterial species. Florida Entomologist 77, 117-126.

Moore, B.P., Brown, W.V., & Rothschild, M. (1990). Methylalkylpyrazines in aposematic insects, their hostplants and mimics. Chemoecology 1, 43–51.

Pettersson, J., Birkett, M.A., & Pickett, J.A. (1999). Pyrazines as attractants for insects of order Coleoptera. International Publication Number WO 99/37152.

Pickering, G.J., Lin, J.Y., Riesen, R., Reynolds, A., Brindle, I., & Soleas, G. (2004). Influence of *Harmonia axyridis* on the sensory properties of white and red wine. America Journal of Enology and Viticulture 55, 153-159.

Pickering, G.J., Lin, Y., Reynolds, A., Soleas, G., Riesen, R., & Brindle, I. (2005). The influence of *Harmonia axyridis* on wine composition and aging. Journal of Food Science 70(2), 128-135.

Pickering, G.J., Spink, M., Kotseridis, Y., Brindle, I.D., Sears, M., & Inglis, D. (2008). The influence of *Harmonia axyridis* morbidity on 2-Isopropyl-3-methoxy-pyrazine in 'Cabernet Sauvignon' wine. Vitis 47(4), 227-230.

Robacker, D.C. (2007). Chemical ecology of bacterial relationships with fruit flies. IOBC/WPRS Bulletin 30(9), 9-22.

Robacker, D.C., & Moreno, D.S. (1995). Protein feeding attenuates attraction of Mexican fruit flies (Diptera: Tephritidae) to volatile bacterial metabolites. Florida Entomologist 78, 497–508.

Robacker, D.C., & Bartelt, R.J. (1997). Chemicals attractive to Mexican fruit fly from *Klebsiella pneumoniae* and *Citrobacter freundii* cultures sampled by solid-phase microextraction. Journal of Chemical Ecology 23, 2897–2915.

Robacker, D.C., & Lauzon, C.R. (2002). Purine metabolizing capability of *Enterobacter agglomerans* affects volatiles production and attractiveness to Mexican fruit fly. Journal of Chemical Ecology 28, 1549-1563.

Robacker, D.C., & Lauzon, C.R., & He, X. (2004). Volatiles production and attractiveness to the Mexican fruit fly of *Enterobacter agglomerans* isolated from apple maggot and Mexican fruit flies. Journal of Chemical Ecology 30, 1329-1347.

Robacker, D.C., Martinez, A.J., Garcia, J.A., & Bartelt, R.J. (1998). Volatiles attractive to the Mexican fruit fly (Diptera: Tephritidae) from eleven bacteria taxa. Florida Entomologist 81, 497-508.

Rothschild, M., Moore, B.P., & Brown, W.V. (1984). Pyrazines as warning odor components in the monarch butterfly, *Danaus plexippus*, and in moths of the genera Zygaena and Amata (Lepidoptera). Biological Journal of Linnean Society 23, 375-380.

Schulz, S., & Dickschat, J.S. (2007). Bacterial volatiles: The smell of small organisms. Natural Product Reports 24, 814-842.

Susset, E.C., Ramon-Portugal. F., Hemptinne, J.L., Dewhirst, S.Y., Birkett, M.A., & Magro, A. (2013). The role of semiochemicals in short-range location of aggregation sites in *Adalia bipunctata* (Coleoptera, Coccinellidae). Journal of Chemical Ecology 39, 591-601.

Tursch, B., Braekman, J.C., Daloze, D., Hootele, C., Losman, D., Karlsson, R., & Pasteels, J.M. (1973). Chemical ecology of arthropods. VI. Adaline, a novel alkaloid from *Adalia bipunctata* L. (Coleoptera, Coccinellidae). Tetrahedron Letters 14, 201-202.

Tursch, B., Daloze, D., Braekman, J.C., Hootele, C., Cravador, A., Losman, D., & Karlsson, R. (1974). Chemical ecology of arthropods. IX. Structure and absolute configuration of hippodamine and convergine, two novel alkaloids from the American ladybug *Hippodamia convergens* (Coleoptera - Coccinellidae). Tetrahedron Letters 15, 409-412.

Tursch, B., Daloze, D., Braekman, J.C., Hootele, C., & Pasteels, J.M. (1975). Chemical ecology of arthropods. X. The structure of myrrhine and the biosynthesis of coccinelline. Tetrahedron Letters 31, 1541-1543.

Verheggen, F.J., Fagel, Q., Heuskin, S., Lognay, G., Francis, F., & Haubruge, E. (2007). Elektrophysiological and behavioral responsens of the multicoloured Asian lady beetle, *Harmonia axyridis* Pallas, to sesquiterpene semiochemicals. Journal of Chemical Ecology 33, 2148-2155.

Wheeler, C.A., & Cardé, R.T. (2013). Defensive allomones function as aggregation pheromones in diapausing ladybird beetles, *Hippodamia convergens*. Journal of Chemical Ecology 39, 723-732.

Zahn, D.K., Moreira, J.A., & Millar, J.G. (2008). Identification, synthesis, and bioassay of male-specific aggregation pheromone from the harlequin bug, *Murgantia histionica*. Journal of Chemical Ecology 34, 238-251.
