# Supplementary material for: Symbiont‐mediated chemical defense in the invasive ladybird Harmonia axyridis
Source: Ecol Evol. 2019 Jan 25;9(4):1715–29. doi: 10.1002/ece3.4840 (PMC6392489; doi:10.1002/ece3.4840)
Supplement: Supplementary file 2 [file ECE3-9-1715-s002.pdf]

Supplementary Material Part II – Statistical Analysis and Data Set

Content:

Figure 1. Methoxypyrazine (MP) content in life stages of the ladybird *H. axyridis* feeding on aphids in greenhouse.

Figure 2. Influence of diets on total MP content in mature beetles.

Figure 4. Influence of antibiotics on total MP content of *H. axyridis*.

Data files for total MP content of diets (aphids and grapes) and tissues (fat body, muscle tissue, hemolymph) of adult beetles.

Data files for SBMP, IPMP, and IBMP content of adult *H. axyridis* feeding on HS and HSAB diet.

Data files for SBMP, IPMP, and IBMP content during development of *H. axyridis* feeding on HS and HSAB diet

Supplementary Material Part II – Statistical Analysis and Data Set

Figure 1. Methoxypyrazine (MP) content in life stages of the ladybird *H. axyridis* feeding on aphids in greenhouse.

Statistical analysis for the different life stages was carried out using SIGMAPLOT v. 12.0 (Systat Software Inc., San Jose, CA). Significant differences between groups of parametric data were determined by one-way analysis of variance (ANOVA) with a subsequent Holm–Sidak test. Non-parametric data were analysed by ANOVA on-ranks with a subsequent Dunn's test.

Fig. 1A. Total MP content

Fig. 1A. Total MP content in life stages

**total MP**		total MP	average total MP	Stdev total MP	total MP	average total MP	Stdev total MP
data file	stage	pg/mg FW	pg/mg FW	pg/mg FW	pg/sample	pg/sample	pg/sample
16_NTD_140407-05_ASW_eggs_1.D	eggs	6,591910557	8,114572955	2,030763935	13,08700625	16,64778738	3,518924794
17_NTD_140407-05_ASW_eggs_2.D	eggs	8,522123811			18,29878139		
18_NTD_140407-05_ASW_eggs_3.D	eggs	9,970540762			15,78892931		
19_NTD_140407-05_ASW_eggs_4.D	eggs	5,475625518			14,18789041		
21_NTD_140407-05_ASW_eggs_5.D	eggs	10,01266413			21,87632953		
11_NTD_140407-05_ASW_L4_1.D	L4	3,433258836	4,19245393	1,398433575	49,46713485	102,4025854	41,03707938
12_NTD_140407-05_ASW_L4_2.D	L4	3,042028394			86,47595481		
13_NTD_140407-05_ASW_L4_3.D	L4	5,705631459			156,4174172		
14_NTD_140407-05_ASW_L4_4.D	L4	5,755791152			146,7031066		
15_NTD_140407-05_ASW_L4_5.D	L4	2,499124946			92,70109074		
05_NTD_140407-05_ASW_L4.D	L4	4,718888789			82,65080835		
27_NTD_140407-05_HA_D_1.D	diapause	13,11506386	23,36394174	10,16452225	341,4802474	235,9427627	112,3490711
28_NTD_140407-05_HA_D_2.D	diapause	31,91307404			308,9604716		
29_NTD_140407-05_HA_D_3.D	diapause	34,88745131			117,4172767		
30_NTD_140407-05_HA_D_4.D	diapause	13,25117294			110,6141247		
31_NTD_140407-05_HA_D_5.D	diapause	23,65294658			301,2416928		
22_NTD_140407-05_ASW_adult_1.D	adult	22,90037446	24,98157932	7,504541784	62,94872397	403,4097479	250,4244593
23_NTD_140407-05_ASW_adult_2.D	adult	14,02819149			238,1761415		
24_NTD_140407-05_ASW_adult_3.D	adult	27,24713378			478,25643		
02_NTD_140407-05_ASW_adult_1.D	adult	26,01606151			550,9485889		
03_NTD_140407-05_ASW_adult_2.D	adult	34,71613532			686,7188554		
29_NTD_140407-005_Ha_3N_1.D	beetle post hatching (p.h.)	19,73428152	22,79763418	5,915375298	83,74622332	120,9038181	54,27836602
30_NTD_140407-005_Ha_3N_2.D	beetle post hatching (p.h.)	16,60790436			82,66119176		
31_NTD_140407-005_Ha_2x_1.D	beetle post hatching (p.h.)	20,16612183			94,28554608		
32_NTD_140407-005_Ha_2x_2.D	beetle post hatching (p.h.)	26,02671211			132,996745		
81_NTD_140407-005_Ha_4.D	beetle post hatching (p.h.)	31,45315111			210,8293843		

Figure 1: MP content in life stages of the ladybird *H. axyridis*.
1A. Total MP content: MP pg/mg fresh weight

One Way Analysis of Variance
Normality Test: Passed (P = 0,137)
Equal Variance Test: Failed (P < 0,050)
Kruskal-Wallis One Way Analysis of Variance on Ranks

Group	*N*	*Missing*	*Median*	*25%*	*75%*
eggs fw	5	0	8,522	6,313	9,981
L4 fw	6	0	4,076	3,042	5,706
beetle p.h. fw	5	0	20,166	18,953	27,383
adult beetle fw	5	0	26,016	20,682	29,114
beetle diapause fw	5	0	23,653	13,217	32,657

H = 19,481 with 4 degrees of freedom. (P = <0,001). The differences in the median values among the treatment groups are greater than would be expected by chance; there is a statistically significant difference (P = <0,001)

All Pairwise Multiple Comparison Procedures (Dunn's Method):

Comparison	*Diff of Ranks*	*Q*	*P<0,05*
adult beetle fw vs L4 fw	15,967	3,447	Yes
adult beetle fw vs eggs fw	11,200	2,315	No
adult beetle vs beetle p.h. f	1,400	0,289	Do Not Test
adult beetle vs beetle diapa	1,000	0,207	Do Not Test
beetle diapause fw vs L4 fw	14,967	3,232	Yes
beetle diapause fw vs eggs fw	10,200	2,109	Do Not Test
beetle diapause vs beetle p.h. fw	0,400	0,0827	Do Not Test
beetle p.h. fw vs L4 fw	14,567	3,145	Yes
beetle p.h. fw vs eggs fw	9,800	2,026	Do Not Test
eggs fw vs L4 fw	4,767	1,029	No

1A. Total MP content: MP pg/sample

One Way Analysis of Variance
Normality Test: Failed (P < 0,050)
Kruskal-Wallis One Way Analysis of Variance on Ranks

Group	*N*	*Missing*	*Median*	*25%*	*75%*
eggs	5	0	15,789	13,913	19,193
L4	6	0	89,589	82,651	146,703
beetle p.h.	5	0	94,286	83,475	152,455
adult beetle	5	0	478,256	194,369	584,891
beetle diapause	5	0	301,242	115,716	317,090

H = 16,309 with 4 degrees of freedom. (P = 0,003)
The differences in the median values among the treatment groups are greater than would be expected by chance; there is a statistically significant difference (P = 0,003)

All Pairwise Multiple Comparison Procedures (Dunn's Method):

Comparison	*Diff of Ranks*	*Q*	*P<0,05*
adult beetle vs eggs	17,400	3,597	Yes
adult beetle vs L4	8,400	1,814	No
adult beetle vs beetle p.h.	7,000	1,447	Do Not Test
adult beetle vs beetle diapause	1,400	0,289	Do Not Test
beetle diapause vs eggs	16,000	3,308	Yes
beetle diapause vs L4	7,000	1,511	Do Not Test
beetle diapause vs beetle p.h.	5,600	1,158	Do Not Test
beetle p.h. vs eggs	10,400	2,150	No
beetle p.h. vs L4	1,400	0,302	Do Not Test
L4 vs eggs	9,000	1,943	Do Not Test

Figure 1. Methoxypyrazine (MP) content in life stages of the ladybird *H. axyridis* feeding on aphids in greenhouse.

Fig. 1B. SBMP content

Fig. 1B. SBMP content in life stages							
**SBMP**		SBMP	average SBMP	Stdev SBMP	SBMP	average SBMP	Stdev SBMP
data file	stage	pg/mg FW	pg/mg FW	pg/mg FW	pg/sample	pg/sample	pg/sample
16_NTD_140407-05_ASW_eggs_1.D	eggs	2,241066585	2,41804082	0,796095053	10,71229827	13,52145248	2,847614737
17_NTD_140407-05_ASW_eggs_2.D	eggs	3,600340937			16,45355808		
18_NTD_140407-05_ASW_eggs_3.D	eggs	1,782277425			11,2818161		
19_NTD_140407-05_ASW_eggs_4.D	eggs	1,670484056			12,49522074		
21_NTD_140407-05_ASW_eggs_5.D	eggs	2,796035099			16,66436919		
11_NTD_140407-05_ASW_L4_1.D	L4	1,949041334	2,3601293	0,752873174	47,7710031	86,10571118	30,14612073
12_NTD_140407-05_ASW_L4_2.D	L4	1,783347015			83,56764113		
13_NTD_140407-05_ASW_L4_3.D	L4	2,099055275			90,80513121		
14_NTD_140407-05_ASW_L4_4.D	L4	3,792672326			138,3187597		
15_NTD_140407-05_ASW_L4_5.D	L4	1,955899498			87,85900544		
05_NTD_140407-05_ASW_L4.D	L4	2,58076035			68,31272645		
27_NTD_140407-05_HA_D_1.D	diapause	8,561463316	5,57043834	2,90648013	322,8527816	224,0867758	110,9698387
28_NTD_140407-05_HA_D_2.D	diapause	8,050028961			290,1230438		
29_NTD_140407-05_HA_D_3.D	diapause	2,484343715			101,6096579		
30_NTD_140407-05_HA_D_4.D	diapause	2,599279775			104,8289533		
31_NTD_140407-05_HA_D_5.D	diapause	6,157075934			301,0194424		
22_NTD_140407-05_ASW_adult_1.D	adult	1,564794521	8,978198092	5,609778529	58,6641466	358,3120681	246,31062
23_NTD_140407-05_ASW_adult_2.D	adult	4,98302302			154,7228648		
24_NTD_140407-05_ASW_adult_3.D	adult	10,76987467			417,5480411		
02_NTD_140407-05_ASW_adult_1.D	adult	12,12743596			511,8990719		
03_NTD_140407-05_ASW_adult_2.D	adult	15,44586228			648,7262159		
29_NTD_140407-005_Ha_3N_1.D	beetle post hatching (p.h.)	3,249185913	4,445689357	1,632620379	83,66653727	118,996902	50,52358916
30_NTD_140407-005_Ha_3N_2.D	beetle post hatching (p.h.)	3,580859265			82,57461466		
31_NTD_140407-005_Ha_2x_1.D	beetle post hatching (p.h.)	3,956049364			94,23309585		
32_NTD_140407-005_Ha_2x_2.D	beetle post hatching (p.h.)	4,141814649			132,8694139		
81_NTD_140407-005_Ha_4.D	beetle post hatching (p.h.)	7,300537596			201,6408484		

Figure 1: MP content in life stages of the ladybird *H. axyridis*.
1B. SBMP content: SBMP pg/mg fresh weight

One Way Analysis of Variance
Normality Test: Passed (P = 0,122)
Equal Variance Test: Failed (P < 0,050)
Kruskal-Wallis One Way Analysis of Variance on Ranks

Group	*N*	*Missing*	*Median*	*25%*	*75%*
eggs fw	5	0	2,241	1,754	2,997
L4 fw	6	0	2,027	1,949	2,581
beetle p.h. fw	5	0	3,956	3,498	4,931
adult beetle fw	5	0	10,770	4,128	12,957
beetle diapause fw	5	0	6,157	2,571	8,178

H = 10,142 with 4 degrees of freedom. (P = 0,038)
The differences in the median values among the treatment groups are greater than would be expected by chance; there is a statistically significant difference (P = 0,038)

All Pairwise Multiple Comparison Procedures (Dunn's Method):

Comparison	*Diff of Ranks*	*Q*	*P<0,05*
adult beetle fw vs L4 fw	11,000	2,375	No
adult beetle fw vs eggs fw	11,000	2,274	Do Not Test
adult beetle vs beetle p.h. f	2,400	0,496	Do Not Test
adult beetle vs beetle diapause	2,000	0,413	Do Not Test
beetle diapause fw vs L4 fw	9,000	1,943	Do Not Test
beetle diapause fw vs eggs fw	9,000	1,861	Do Not Test
beetle diapau vs beetle p.h. f	0,400	0,0827	Do Not Test
beetle p.h. fw vs L4 fw	8,600	1,857	Do Not Test
beetle p.h. fw vs eggs fw	8,600	1,778	Do Not Test
eggs fw vs L4 fw	0,000	0,000	Do Not Test

1B. SBMP content: SBMP pg/sample

One Way Analysis of Variance
Normality Test: Failed (P < 0,050)
Kruskal-Wallis One Way Analysis of Variance on Ranks

Group	*N*	*Missing*	*Median*	*25%*	*75%*
eggs	5	0	12,495	11,139	16,506
L4	6	0	85,713	68,313	90,805
beetle p.h.	5	0	94,233	83,394	150,062
adult beetle	5	0	417,548	130,708	546,106
beetle diapause	5	0	290,123	104,024	306,478

H = 16,835 with 4 degrees of freedom. (P = 0,002)
The differences in the median values among the treatment groups are greater than would be expected by chance; there is a statistically significant difference (P = 0,002)

All Pairwise Multiple Comparison Procedures (Dunn's Method):

Comparison	*Diff of Ranks*	*Q*	*P<0,05*
adult beetle vs eggs	17,200	3,556	Yes
adult beetle vs L4	9,033	1,950	No
adult beetle vs beetle p.h.	6,000	1,240	Do Not Test
adult beetle vs beetle diapause	0,800	0,165	Do Not Test
beetle diapause vs eggs	16,400	3,390	Yes
beetle diapause vs L4	8,233	1,778	Do Not Test
beetle diapause vs beetle p.h.	5,200	1,075	Do Not Test
beetle p.h. vs eggs	11,200	2,315	No
beetle p.h. vs L4	3,033	0,655	Do Not Test
L4 vs eggs	8,167	1,763	Do Not Test

Figure 1. Methoxypyrazine (MP) content in life stages of the ladybird *H. axyridis* feeding on aphids in greenhouse.

Fig. 1C. IPMP content

Fig. 1C. IPMP content in life stages							
**IPMP**		IPMP	average IPMP	Stdev IPMP	IPMP	average IPMP	Stdev IPMP
data file	stage	pg/mg FW	pg/mg FW	pg/mg FW	pg/sample	pg/sample	pg/sample
16_NTD_140407-05_ASW_eggs_1.D	eggs	3,862573314	5,162708927	1,687027767	0,040774222	0,051925185	0,01354881
17_NTD_140407-05_ASW_eggs_2.D	eggs	4,525790728			0,035539201		
18_NTD_140407-05_ASW_eggs_3.D	eggs	7,484732775			0,053764752		
19_NTD_140407-05_ASW_eggs_4.D	eggs	3,587773616			0,066758182		
21_NTD_140407-05_ASW_eggs_5.D	eggs	6,3526742			0,062789568		
11_NTD_140407-05_ASW_L4_1.D	L4	1,421404409	1,413535096	0,565176899	0,156582845	0,078926306	0,043958893
12_NTD_140407-05_ASW_L4_2.D	L4	1,198874054			0,105742405		
13_NTD_140407-05_ASW_L4_3.D	L4	2,091154699			0,055152639		
14_NTD_140407-05_ASW_L4_4.D	L4	1,734426328			0,043931416		
15_NTD_140407-05_ASW_L4_5.D	L4	0,436541192			0,049828508		
05_NTD_140407-05_ASW_L4.D	L4	1,598809895			0,062320021		
27_NTD_140407-05_HA_D_1.D	diapause	4,062604788	17,48632571	10,88983083	0,112015735	0,128090212	0,058533015
28_NTD_140407-05_HA_D_2.D	diapause	23,34205592			0,060978513		
29_NTD_140407-05_HA_D_3.D	diapause	32,01967944			0,125407506		
30_NTD_140407-05_HA_D_4.D	diapause	10,51141777			0,119798892		
31_NTD_140407-05_HA_D_5.D	diapause	17,49587064			0,222250414		
22_NTD_140407-05_ASW_adult_1.D	adult	21,22274065	14,76881648	5,670113625	0,054232337	0,192178356	0,237171463
23_NTD_140407-05_ASW_adult_2.D	adult	6,359399928			0,060163401		
24_NTD_140407-05_ASW_adult_3.D	adult	14,92720767			0,612894638		
02_NTD_140407-05_ASW_adult_1.D	adult	12,96649165			0,126244967		
03_NTD_140407-05_ASW_adult_2.D	adult	18,36824249			0,107356439		
29_NTD_140407-005_Ha_3N_1.D	beetle post hatching (p.h.)	16,48509561	18,28593879	4,438895031	0,079686055	0,083829336	0,027462868
30_NTD_140407-005_Ha_3N_2.D	beetle post hatching (p.h.)	13,02704509			0,086577094		
31_NTD_140407-005_Ha_2x_1.D	beetle post hatching (p.h.)	16,21007247			0,052450236		
32_NTD_140407-005_Ha_2x_2.D	beetle post hatching (p.h.)	21,88489746			0,127331068		
81_NTD_140407-005_Ha_4.D	beetle post hatching (p.h.)	23,82258333			0,073102228		

Figure 1: MP content in life stages of the ladybird *H. axyridis*.
1C. IPMP content: IPMP pg/mg fresh weight

One Way Analysis of Variance
Normality Test: Passed (P = 0,079)
Equal Variance Test: Failed (P < 0,050)
Kruskal-Wallis One Way Analysis of Variance on Ranks

Group	*N*	*Missing*	*Median*	*25%*	*75%*
eggs fw	5	0	4,526	3,794	6,636
L4 fw	6	0	1,510	1,199	1,734
beetle p.h. fw	5	0	16,485	15,414	22,369
adult beetle fw	5	0	14,927	11,315	19,082
beetle diapause fw	5	0	17,496	8,899	25,511

H = 18,786 with 4 degrees of freedom. (P = <0,001)
The differences in the median values among the treatment groups are greater than would be expected by chance; there is a statistically significant difference (P = <0,001)

All Pairwise Multiple Comparison Procedures (Dunn's Method):

Comparison	*Diff of Ranks*	*Q*	*P<0,05*
beetle p.h. fw vs L4 fw	16,700	3,606	Yes
beetle p.h. fw vs eggs fw	10,400	2,150	No
beetle p.h. fw vs adult beetle fw	2,800	0,579	Do Not Test
beetle p.h. fw vs beetle diapause fw	1,600	0,331	Do Not Test
beetle diapause fw vs L4 fw	15,100	3,260	Yes
beetle diapause fw vs eggs fw	8,800	1,819	Do Not Test
beetle diapause fw vs adult beetle fw	1,200	0,248	Do Not Test
adult beetle fw vs L4 fw	13,900	3,001	Yes
adult beetle fw vs eggs fw	7,600	1,571	Do Not Test
eggs fw vs L4 fw	6,300	1,360	No

1C. IPMP content: IPMP pg/mg sample

One Way Analysis of Variance
Normality Test: Failed (P < 0,050)
Kruskal-Wallis One Way Analysis of Variance on Ranks

Group	*N*	*Missing*	*Median*	*25%*	*75%*
eggs	5	0	0,0538	0,0395	0,0638
L4	6	0	0,0587	0,0498	0,106
beetle p.h.	5	0	0,0797	0,0679	0,0968
adult beetle	5	0	0,107	0,0587	0,248
beetle diapause	5	0	0,120	0,0993	0,150

H = 7,803 with 4 degrees of freedom. (P = 0,099)
The differences in the median values among the treatment groups are not great enough to exclude the possibility that the difference is due to random sampling variability; there is not a statistically significant difference

Figure 1. Methoxypyrazine (MP) content in life stages of the ladybird *H. axyridis* feeding on aphids in greenhouse.

Fig. 1D. IBMP content

Fig. 1D. IBMP content in life stages							
**IBMP**		IBMP	average IBMP	Stdev IBMP	IBMP	average IBMP	Stdev IBMP
data file	stage	pg/mg FW	pg/mg FW	pg/mg FW	pg/sample	pg/sample	pg/sample
16_NTD_140407-05_ASW_eggs_1.D	eggs	0,488270659	0,533823208	0,254462131	2,333933749	3,074409714	1,616484468
17_NTD_140407-05_ASW_eggs_2.D	eggs	0,395992146			1,809684107		
18_NTD_140407-05_ASW_eggs_3.D	eggs	0,703530562			4,453348459		
19_NTD_140407-05_ASW_eggs_4.D	eggs	0,217367846			1,625911484		
21_NTD_140407-05_ASW_eggs_5.D	eggs	0,863954827			5,149170772		
11_NTD_140407-05_ASW_L4_1.D	L4	0,062813093	0,418789534	0,566791481	1,539548912	16,21794795	24,60145107
12_NTD_140407-05_ASW_L4_2.D	L4	0,059807325			2,80257127		
13_NTD_140407-05_ASW_L4_3.D	L4	1,515421484			65,5571334		
14_NTD_140407-05_ASW_L4_4.D	L4	0,228692499			8,34041544		
15_NTD_140407-05_ASW_L4_5.D	L4	0,106684256			4,792256793		
05_NTD_140407-05_ASW_L4.D	L4	0,539318544			14,27576187		
27_NTD_140407-05_HA_D_1.D	diapause	0,490995758	0,307177691	0,227742371	18,51545004	11,72789663	8,451143992
28_NTD_140407-05_HA_D_2.D	diapause	0,52098916			18,77644932		
29_NTD_140407-05_HA_D_3.D	diapause	0,383428149			15,68221129		
30_NTD_140407-05_HA_D_4.D	diapause	0,14047539			5,665372492		
31_NTD_140407-05_HA_D_5.D	diapause	0			0		
22_NTD_140407-05_ASW_adult_1.D	adult	0,112839291	1,234564745	0,958121122	4,230345026	44,90550152	29,37503632
23_NTD_140407-05_ASW_adult_2.D	adult	2,685768546			83,39311334		
24_NTD_140407-05_ASW_adult_3.D	adult	1,550051437			60,09549421		
02_NTD_140407-05_ASW_adult_1.D	adult	0,922133902			38,92327202		
03_NTD_140407-05_ASW_adult_2.D	adult	0,902030548			37,88528301		
29_NTD_140407-005_Ha_3N_1.D	beetle post hatching (p.h.)	0	0,066006037	0,147593986	0	1,823086743	4,076545887
30_NTD_140407-005_Ha_3N_2.D	beetle post hatching (p.h.)	0			0		
31_NTD_140407-005_Ha_2x_1.D	beetle post hatching (p.h.)	0			0		
32_NTD_140407-005_Ha_2x_2.D	beetle post hatching (p.h.)	0			0		
81_NTD_140407-005_Ha_4.D	beetle post hatching (p.h.)	0,330030185			9,115433717		

Figure 1: MP content in life stages of the ladybird *H. axyridis*.
1D. IBMP content: IBMP pg/mg fresh weight

One Way Analysis of Variance
Normality Test: Failed (P < 0,050)
Kruskal-Wallis One Way Analysis of Variance on Ranks

Group	*N*	*Missing*	*Median*	*25%*	*75%*
Eggs fw	5	0	0,488	0,351	0,744
L4 fw	6	0	0,168	0,0628	0,539
Beetle p.h. fw	5	0	0,000	0,000	0,0825
Adult beetle fw	5	0	0,922	0,705	1,834
Beetle diapause fw	5	0	0,383	0,105	0,498

H = 12,061 with 4 degrees of freedom. (P = 0,017)
The differences in the median values among the treatment groups are greater than would be expected by chance; there is a statistically significant difference (P = 0,017)

All Pairwise Multiple Comparison Procedures (Dunn's Method):

Comparison	*Diff of Ranks*	*Q*	*P<0,05*
Adult beetle fw vs Beetle p.h. fw	16,000	3,308	Yes
Adult beetle fw vs Beetle diapause fw	8,600	1,778	No
Adult beetle fw vs L4 fw	8,333	1,799	Do Not Test
Adult beetle fw vs Eggs fw	4,400	0,910	Do Not Test
Eggs fw vs Beetle p.h. fw	11,600	2,398	No
Eggs fw vs Beetle diapause fw	4,200	0,868	Do Not Test
Eggs fw vs L4 fw	3,933	0,849	Do Not Test
L4 fw vs Beetle p.h. fw	7,667	1,655	Do Not Test
L4 fw vs Beetle diapause fw	0,267	0,0576	Do Not Test
Beetle diapause fw vs Beetle p.h. fw	7,400	1,530	Do Not Test

1D. IBMP content: IBMP pg/sample

One Way Analysis of Variance
Normality Test: Failed (P < 0,050)
Kruskal-Wallis One Way Analysis of Variance on Ranks

**Group**	**N**	**Missing**	**Median**	**25%**	**75%**
eggs	5	0	2,334	1,764	4,627
L4	6	0	6,566	2,803	14,276
Beetle p.h.	5	0	0,000	0,000	2,279
Adult beetle	5	0	38,923	29,472	65,920
Beetle diapause	5	0	15,682	4,249	18,581

H = 11,779 with 4 degrees of freedom. (P = 0,019)
The differences in the median values among the treatment groups are greater than would be expected by chance; there is a statistically significant difference (P = 0,019)

All Pairwise Multiple Comparison Procedures (Dunn's Method):

Comparison	*Diff of Ranks*	*Q*	*P<0,05*
Adult beetle vs Beetle p.h.	15,400	3,184	Yes
Adult beetle vs eggs	11,200	2,315	No
Adult beetle vs L4	6,533	1,411	Do Not Test
Adult beetle vs Beetle diapause	5,600	1,158	Do Not Test
Beetle diapause vs Beetle p.h.	9,800	2,026	No
Beetle diapause vs eggs	5,600	1,158	Do Not Test
Beetle diapause vs L4	0,933	0,202	Do Not Test
L4 vs Beetle p.h.	8,867	1,914	Do Not Test
L4 vs eggs	4,667	1,008	Do Not Test
eggs vs Beetle p.h.	4,200	0,868	Do Not Test

Figure 2. Influence of diets on total MP content in mature beetles. Data were collected from dissected guts (gut) and rest-body tissue (body) of male and female *H. axyrids.* The following feeding assays were performed for 10 days:

Statistical analysis for the different diets was carried out using SIGMAPLOT v. 12.0 (Systat Software Inc., San Jose, CA). Significant differences between groups of parametric data were determined by one-way analysis of variance (ANOVA) with a subsequent Holm–Sidak test. Non-parametric data were analysed by ANOVA on-ranks with a subsequent Dunn's test.

Fig. 2A. Aphid diet on Petri dishes.

Figure 2: aphid diet in Petri dish, female vs. male, gut vs. residual body							
data File		total MP pg/mg FW	average total MP pg/mg FW	Stdev total MP pg/mg FW	total MP pg/sample	average total MP pg/sample	Stdev total MP pg/sample
04_NTD140407-005_160121_F1g.D	female gut	9,462549574	5,78277743	3,62655552	35,4845609	20,0131307	9,946972963
08_NTD140407-005_160121_F2g.D	female gut	3,340969337			17,07235331		
21_NTD140407-005_160121_F5g.D	female gut	3,572895			14,29158		
39_NTD140407-05_160216_FX12g.D	female gut	9,993557217			23,38492389		
37_NTD140407-006_160121_F6-11g.D	female gut	2,543916023			9,83223542		
05_NTD140407-005_160121_F1b.D	female residual body	19,31066423	8,514065355	4,54475751	428,3105327	205,9076729	109,3388053
09_NTD140407-005_160121_F2b.D	female residual body	9,461916677			216,5832727		
14_NTD140407-005_160121_F3b.D	female residual body	6,391841137			143,5607519		
22_NTD140407-005_160121_F5b.D	female residual body	8,056055003			180,5361926		
18_NTD140407-005_160121_F4b.D	female residual body	6,259855352			165,5105755		
25_NTD140407-005_160121_F6b.D	female residual body	4,530295343			128,8415996		
28_NTD140407-006_160121_F7b.D	female residual body	3,275455544			63,87138312		
32_NTD140407-006_160121_F9b.D	female residual body	7,291644489			174,2703033		
34_NTD140407-006_160121_F10b.D	female residual body	8,334903224			194,7866883		
36_NTD140407-006_160121_F11b.D	female residual body	12,22802255			362,805429		
06_NTD140407-005_160121_M1g.D	male gut	24,01613391	11,95588536	8,387461104	60,04033478	30,63964259	22,98098039
10_NTD140407-005_160121_M2g.D	male gut	3,199143751			13,75631813		
15_NTD140407-005_160121_M3g.D	male gut	17,91399861			59,83275536		
19_NTD140407-005_160121_M4g.D	male gut	15,24693243			19,36360419		
23_NTD140407-005_160121_M5g.D	male gut	4,179123769			10,48960066		
38_NTD140407-006_160121_M6g.D	male gut	7,179979686			20,35524242		
07_NTD140407-005_160121_M1b.D	male residual body	18,93785344	22,38550144	10,04641269	390,498538	426,4782981	198,4839467
11_NTD140407-005_160121_M2b.D	male residual body	17,72949733			361,5044505		
20_NTD140407-005_160121_M4b.D	male residual body	13,76614759			255,0867148		
24_NTD140407-005_160121_M5b.D	male residual body	15,21278626			272,6131298		
26_NTD140407-005_160121_M6b.D	male residual body	37,77396062			759,2566084		
29_NTD140407-006_160121_M7b.D	male residual body	12,34950602			253,7823486		
30_NTD140407-006_160121_M8b.D	male residual body	27,31230426			415,9663939		
35_NTD140407-006_160121_M10b.D	male residual body	36,00195601			703,1182009		

Statistical analysis

Figure 2: Influence of diets on total MP content

2A. Aphid diet: MP pg/mg fresh weight

One Way Analysis of Variance

Normality Test: Passed (P = 0,087)

Equal Variance Test: Passed (P = 0,096)

Groups	*N*	*Missing*	*Mean*	*Std Dev*	*SEM*
female gut fw	5	0	5,782	3,626	1,593
male gut fw	6	0	11,956	8,387	3,424
female body fw	10	0	8,514	4,545	1,437
male body fw	8	0	22,386 1	10,046	3,552

The differences in the mean values among the treatment groups are greater than would be expected by chance; there is a statistically significant difference (P = 0,001).

Power of performed test with alpha = 0,050: 0,947

All Pairwise Multiple Comparison Procedures (Holm-Sidak method): Overall significance level = 0,05

Comparison	*Diff of Means*	*t*	*Unadjusted P*	*Critical Level*	*Significant?*
male body fw vs. female body fw	13,871	4,013	<0,001	0,009	Yes
male body fw vs. female gut fw	17,655	3,956	<0,001	0,010	Yes
male body fw vs. male gut fw	10,430	2,650	0,014	0,013	No
male gut fw vs. female gut fw	7,226	1,536	0,138	0,017	No
male gut fw vs. female body fw	3,442	0,915	0,370	0,025	No
female body fw vs. female gut fw	3,784	0,878	0,389	0,050	No

2A. Aphid diet: MP pg/sample

One Way Analysis of Variance

Normality Test: Failed (P < 0,050)

Kruskal-Wallis One Way Analysis of Variance on Ranks

Group	*N*	*Missing*	*Median*	*25%*	*75%*
female gut sample	4	0	15,682	12,062	26,278
male gut sample	6	0	19,859	13,756	59,833
female body sample	10	0	177,403	143,561	216,583
male body sample	8	0	376,001	263,850	559,542

H = 21,712 with 3 degrees of freedom. (P = <0,001).

The differences in the median values among the treatment groups are greater than would be expected by chance; there is a statistically significant difference (P = <0,001)

All Pairwise Multiple Comparison Procedures (Dunn's Method):

Comparison	*Diff of Ranks*	*Q*	*P<0,05*
male body vs female gut	18,750	3,722	Yes
male body vs male gut	17,083	3,845	Yes
male body vs female body	6,750	1,730	No
female body vs female gut	12,000	2,466	No
female body vs male gut	10,333	2,433	Do Not Test
male gut vs female gut	1,667	0,314	Do Not Test

Figure 2. Influence of diets on total MP content in mature beetles.

Figure 2B. Grape diet on Petri dishes.

Figure 2: grape diet in Petri dish, female vs. male, gut vs. residual body

Data File	stage	total MP pg/mg FW	average total MP pg/mg FW	Stdev total MP pg/mg FW	total MP pg/sample	average total MP pg/sample	Stdev total MP pg/sample
02_NTD140407-007_160201_F1g.D	female gut 1	6,557362015	5,741454629	2,404334073	30,22943889	25,41394911	12,74173105
10_NTD140407-007_160201_F3g.D	female gut 3	3,64913717			12,73548872		
15_NTD140407-008_160201_F4g.D	female gut 4	9,262698494			44,9240877		
19_NTD140407-008_160201_F5g.D	female gut 5	3,361859466			16,67482295		
34_NTD140407-009_160201_F6g.D	female gut 6	5,876216003			22,5059073		
03_NTD140407-007_160201_F1b.D	female residual body 1	12,012075	14,89777936	8,323611043	315,677331	390,0811978	263,8747798
11_NTD140407-007_160201_F3b.D	female residual body 3	14,13260971			282,369542		
16_NTD140407-008_160201_F4b.D	female residual body 4	27,96346305			787,7307541		
20_NTD140407-008_160201_F5b.D	female residual body 5	11,27223506			275,0425354		
23_NTD140407-008_160201_F6b.D	female residual body 6	12,88875972			347,4809622		
26_NTD140407-009_160201_F7b.D	female residual body 7	12,28500776			292,2603346		
28_NTD140407-009_160201_F8b.D	female residual body 8	6,054251206			153,8385232		
30_NTD140407-009_160201_F9b.D	female residual body 9	29,75757895			890,9419139		
32_NTD140407-009_160201_F10b.D	female residual body 10	7,714033764			165,3888839		
04_NTD140407-007_160201_M1g.D	male gut 1	13,13541263	7,253423522	3,456070885	25,35134637	17,88791802	7,293297204
08_NTD140407-007_160201_M2g.D	male gut 2	6,474489156			15,02081484		
12_NTD140407-007_160201_M3g.D	male gut 3	7,536026366			24,94424727		
17_NTD140407-008_160201_M4g.D	male gut 4	2,460536513			5,56081252		
21_NTD140407-008_160201_M5g.D	male gut 5	6,153833243			18,3999614		
35_NTD140407-009_160201_M6-10g.D	male gut 6	7,760243226			18,05032574		
05_NTD140407-007_160201_M1b.D	male residual body 1	16,13181318	11,7172768	5,685082885	430,0741393	272,1088769	140,0252257
09_NTD140407-007_160201_M2b.D	male residual body 2	20,88788811			468,3064515		
13_NTD140407-007_160201_M3b.D	male residual body 3	15,99619367			372,3913887		
18_NTD140407-008_160201_M4b.D	male residual body 4	17,44570446			387,9924673		
22_NTD140407-008_160201_M5b.D	male residual body 5	5,830564597			127,3978365		
24_NTD140407-008_160201_M6b.D	male residual body 6	13,22606793			308,4319042		
27_NTD140407-009_160201_M7b.D	male residual body 7	2,58463313			49,31480011		
29_NTD140407-009_160201_M8b.D	male residual body 8	13,26354991			250,5484578		
31_NTD140407-009_160201_M9b.D	male residual body 9	9,617758427			222,55493		
33_NTD140407-009_160201_M10b.D	male residual body 10	9,7149395			195,1731345		
36_NTD140407-009_160201_M11b.D	male residual body 11	3,407493135			78,4745669		
37_NTD140407-009_160201_M12b.D	male residual body 12	12,50071558			374,6464459		

Figure 2: Influence of diets on total MP content

2B. Grape diet: MP pg/mg fresh weight

One Way Analysis of Variance

Normality Test: Passed (P = 0,109)

Equal Variance Test: Passed (P = 0,359)

Group Name	*N*	*Missing*	*Mean*	*Std Dev*	*SEM*
female gut fw	5	0	5,741	2,404	1,075
male gut fw	6	0	7,253	3,456	1,411
female body fw	9	0	14,898	8,324	2,775
male body fw	12	0	11,717	5,685	1,641

The differences in the mean values among the treatment groups are greater than would be expected by chance; there is a statistically significant difference (P = 0,031).

Power of performed test with alpha = 0,050: 0,538

All Pairwise Multiple Comparison Procedures (Holm-Sidak method): Overall significance level = 0,05

Comparison	*Diff of Means*	*t*	*Unadjusted P*	*Critical Level*	*Significant?*
female body fw vs. female gut fw	9,156	2,757	0,010	0,009	No
female body fw vs. male gut fw	7,644	2,436	0,021	0,010	No
male body fw vs. female gut fw	5,976	1,886	0,070	0,013	No
male body fw vs. male gut fw	4,464	1,499	0,145	0,017	No
female body fw vs. male body fw	3,181	1,211	0,236	0,025	No
male gut fw vs. female gut fw	1,512	0,419	0,678	0,050	No

2B. Grape diet: MP pg/sample

One Way Analysis of Variance

Normality Test: Failed (P < 0,050)

Kruskal-Wallis One Way Analysis of Variance on Ranks

Group	*N*	*Missing*	*Median*	*25%*	*75%*
female gut	5	0	22,506	15,690	33,903
male gut	6	0	18,225	15,021	24,944
female body	9	0	292,260	247,629	457,543
male body	12	0	279,490	161,285	381,319

H = 21,208 with 3 degrees of freedom. (P = <0,001)

The differences in the median values among the treatment groups are greater than would be expected by chance; there is a statistically significant difference (P = <0,001)

All Pairwise Multiple Comparison Procedures (Dunn's Method):

Comparison	*Diff of Ranks*	*Q*	*P<0,05*
female body vs male gut	17,556	3,551	Yes
female body vs female gut	16,089	3,075	Yes
female body vs male body	1,556	0,376	No
male body vs male gut	16,000	3,411	Yes
male body vs female gut	14,533	2,911	Yes
female gut vs male gut	1,467	0,258	No

Figure 2. Influence of diets on total MP content in mature beetles.

Figure 2C. Honey syrup diet on Petri dishes.

Figure 2: honey syrup diet in Petri dish, female vs. male, gut vs. residual body							
Data File	stage	total MP pg/mg FW	average total MP pg/mg FW	Stdev total MP pg/mg FW	total MP pg/sample	average total MP pg/sample	Stdev total MP pg/sample
04_NTD140407-010_160203_HS-F2g.D	female gut	32,79064402	21,48739381	7,613028602	20,65810573	33,6037669	12,09117791
12_NTD140407-010_160203_HS-F4g.D	female gut	28,68114425			50,76562531		
21_NTD140407-011_160203_HS-F5g.D	female gut	14,98973103			21,13552075		
29_NTD140407-011_160203_HS-F10g.D	female gut	15,96214502			31,92429004		
42_NTD140407-011_160204_HS-F12g.D	female gut	30,12911036			48,20657658		
47_NTD140407-012_160204_HS-F13g.D	female gut	17,36453946			42,89041247		
38_NTD140407-011_160204_HS-F11g.D	female gut	16,8113581			24,88080999		
51_NTD140407-012_160204_HS-F14g.D	female gut	15,17047825			28,36879433		
05_NTD140407-010_160203_HS-F2b.D	female residual body	25,01603046	16,47644496	4,484824497	666,4270515	386,0583795	148,0424521
13_NTD140407-010_160203_HS-F4b.D	female residual body	17,61576024			434,9331202		
22_NTD140407-011_160203_HS-F5b.D	female residual body	12,20427183			311,3309744		
30_NTD140407-011_160203_HS-F10b.D	female residual body	12,04256624			210,2632066		
43_NTD140407-011_160204_HS-F12b.D	female residual body	18,4273322			449,0740856		
48_NTD140407-012_160204_HS-F13b.D	female residual body	15,49858283			314,7762172		
53_NTD140407-013_160204_HS-F14b.D	female residual body	14,53057094			315,6040008		
08_NTD140407-010_160203_HS-M1g.D	male gut	6,1323953	15,38374118	6,935936416	13,12332594	18,31236992	7,354497018
16_NTD140407-010_160203_HS-M3g.D	male gut	23,74154803			15,90683718		
25_NTD140407-011_160203_HS-M6g.D	male gut	8,810876949			11,71846634		
34_NTD140407-011_160204_HS-M7g.D	male gut	18,67930376			14,00947782		
44_NTD140407-011_160204_HS-M8g.D	male gut	20,81090514			26,01363143		
49_NTD140407-012_160204_HS-M9g.D	male gut	14,12741787			29,10248082		
09_NTD140407-010_160203_HS-M1b.D	male residual body	16,87476602	20,89676167	4,38650019	383,394684	436,0804493	104,1852431
17_NTD140407-010_160203_HS-M3b.D	male residual body	19,78322617			376,8704585		
26_NTD140407-011_160203_HS-M6b.D	male residual body	15,13850544			294,7467009		
35_NTD140407-011_160204_HS-M7b.D	male residual body	22,94855282			455,5287735		
45_NTD140407-011_160204_HS-M8b.D	male residual body	24,23778328			540,5025672		
50_NTD140407-012_160204_HS-M9b.D	male residual body	26,39773632			565,4395119		

Figure 2: Influence of diets on total MP content

2C. Honey syrup diet: MP pg/mg fresh weight

One Way Analysis of Variance
Normality Test: Passed (P = 0,130)
Equal Variance Test: Passed (P = 0,694)

Group Name	*N*	*Missing*	*Mean*	*Std Dev*	*SEM*
female gut fw	8	0	21,487	7,613	2,692
male gut fw	6	0	15,384	6,936	2,832
female body fw	7	0	16,476	4,485	1,695
male body fw	6	0	20,897	4,387	1,791

The differences in the mean values among the treatment groups are not great enough to exclude the possibility that the difference is due to random sampling variability; there is not a statistically significant difference (P = 0,194).
Power of performed test with alpha = 0,050: 0,171. The power (0,171) is below the desired power of 0,800.

2C. Honey syrup diet: MP pg/sample

One Way Analysis of Variance
Normality Test: Failed (P < 0,050)
Kruskal-Wallis One Way Analysis of Variance on Ranks

Group	*N*	*Missing*	*Median*	*25%*	*75%*
female gut sample	8	0	30,147	23,008	45,548
male gut sample	6	0	14,958	13,123	26,014
female body sample	7	0	315,604	312,192	445,539
male body sample	6	0	419,462	376,870	540,503

H = 21,015 with 3 degrees of freedom. (P = <0,001)
The differences in the median values among the treatment groups are greater than would be expected by chance; there is a statistically significant difference (P = <0,001)

All Pairwise Multiple Comparison Procedures (Dunn's Method):

Comparison	*Diff of Ranks*	*Q*	*P<0,05*
male body vs male gut	17,333	3,782	Yes
male body vs female gut	12,375	2,887	Yes
male body vs female body	1,857	0,421	No
female body vs male gut	15,476	3,505	Yes
female body vs female gut	10,518	2,560	No
female gut vs male gut	4,958	1,157	No

Figure 2. Influence of diets on total MP content in mature beetles.

Figure 2D. Honey syrup-*Sitotroga* eggs diet on Petri dishes.

Figure 2: honey syrup- Sitotroga eggs diet in Petri dish, female vs. male, gut vs. residual body							
Data File	stage	total MP pg/mg FW	average total MP pg/mg FW	Stdev total MP pg/mg FW	total MP pg/sample	average total MP pg/sample	Stdev total MP pg/sample
21_NTD140407-05_160216_HSS-F10g.D	female gut	7,026077719	9,73494517	5,658094103	30,91474196	31,67766414	23,71922933
23_NTD140407-05_160216_HSS-F11g.D	female gut	8,806125716			11,97633097		
44_NTD140407-05_160216_HSS-F3g.D	female gut	8,146713093			23,7069351		
77_NTD140407-05_160216_HSS-F5g.D	female gut	16,8642328			60,03666878		
84_NTD140407-05_160216_HSS-F17g.D	female gut	17,99448879			68,1991125		
88_NTD140407-05_160216_HSS-F7g.D	female gut	6,96344876			21,23851872		
82_NTD140407-05_160216_HSS-F16g.D	female gut	2,343529308			5,671340926		
22_NTD140407-05_160216_HSS-F10b.D	female residual body	19,07091313	19,25125866	5,977344766	923,2229047	643,6887197	267,4495784
24_NTD140407-05_160216_HSS-F11b.D	female residual body	13,42909779			298,125971		
45_NTD140407-05_160216_HSS-F3b.D	female residual body	15,03873442			423,3403739		
78_NTD140407-05_160216_HSS-F5b.D	female residual body	30,75661731			955,3005335		
85_NTD140407-05_160216_HSS-F17b.D	female residual body	21,4717987			694,3979699		
87_NTD140407-05_160216_HSS-F6b.D	female residual body	20,59657354			806,3558542		
89_NTD140407-05_160216_HSS-F7b.D	female residual body	14,39507572			405,0774307		
16_NTD140407-05_160216_HSS-M18g.D	male gut	11,32501308	16,88674067	14,66062166	17,78027053	20,67521421	13,5439846
25_NTD140407-05_160216_HSS-M1g.D	male gut	44,12361504			44,56485119		
33_NTD140407-05_160216_HSS-M2g.D	male gut	18,49936474			16,83442191		
42_NTD140407-05_160216_HSS-M13g.D	male gut	17,63944626			27,16474724		
54_NTD140407-05_160216_HSS-M4g.D	male gut	7,074399868			7,923327852		
80_NTD140407-05_160216_HSS-M15g.D	male gut	2,65860504			9,783666548		
17_NTD140407-05_160216_HSS-M18b.D	male residual body	2,474163107	25,62961144	15,39791959	55,96556947	530,7803954	312,0100483
26_NTD140407-05_160216_HSS-M1b.D	male residual body	37,52370959			856,2910528		
34_NTD140407-05_160216_HSS-M2b.D	male residual body	32,43926381			623,4826505		
43_NTD140407-05_160216_HSS-M13b.D	male residual body	34,14352586			689,0163518		
55_NTD140407-05_160216_HSS-M4b.D	male residual body	9,801286486			238,5633131		
81_NTD140407-05_160216_HSS-M15b.D	male residual body	37,39571979			721,3634347		

Figure 2: Influence of diets on total MP content

2D. Honey syrup-*Sitotroga* eggs diet: MP pg/mg fresh weight

One Way Analysis of Variance
Normality Test: Passed (P = 0,724)
Equal Variance Test: Passed (P = 0,502)

Group Name	N	Missing	Mean	Std Dev	SEM
female gut fw	7	0	9,735	5,658	2,139
male gut fw	6	0	16,887	14,661	5,985
female body fw	7	0	19,251	5,977	2,259
male body fw	6	0	25,630	15,398	6,286

The differences in the mean values among the treatment groups are not great enough to exclude the possibility that the difference is due to random sampling variability; there is not a statistically significant difference (P = 0,104).
Power of performed test with alpha = 0,050: 0,294. The power (0,294) is below the desired power of 0,800.

2C. Honey syrup-*Sitotroga* eggs diet: MP pg/sample

One Way Analysis of Variance
Normality Test: Passed (P = 0,069)
Equal Variance Test: Failed (P < 0,050)
Kruskal-Wallis One Way Analysis of Variance on Ranks

Group	N	Missing	Median	25%	75%
female gut	6	0	22,473	11,976	60,037
male gut	6	0	17,307	9,784	27,165
female body	7	0	694,398	409,643	894,006
male body	6	0	656,250	238,563	721,363

H = 17,529 with 3 degrees of freedom. (P = <0,001)
The differences in the median values among the treatment groups are greater than would be expected by chance; there is a statistically significant difference (P = <0,001)

All Pairwise Multiple Comparison Procedures (Dunn's Method):

Comparison	Diff of Ranks	Q	P<0,05
female body vs male gut	14,024	3,425	Yes
female body vs female gut	12,357	3,018	Yes
female body vs male body	2,190	0,535	No
male body vs male gut	11,833	2,785	Yes
male body vs female gut	10,167	2,393	No
female gut vs male gut	1,667	0,392	No

Figure 4. Influence of antibiotics on total MP content of *H. axyridis*.

A. Statistical analysis for Figure 4

For the pairwise comparison of controls and antibiotic treatments we used IBM SPSS Statistics v23 software (Armonk, USA). Statistical differences were calculated by Mann-Whitney-U test for non-parametric data and Student's t-test for normally distributed data.

Figure 4A. Total MP concentration in pg/mg fresh weight of dissected guts (gut) and residual bodies (body) of female and male *H. axyridis* fed for 10 days with *Sitotroga* eggs (in honey) and *Sitotroga* eggs mixed with honey and antibiotics. There are no signifivantl differences for the pairwise comparison of control individuals and the respective antibiotic-treated individuals and tissues.

Figure 4B. Total MP contents in pg/sample after same procedure as shown in 4A. There are no significant differences for the pairwise comparison of control individuals and the respective antibiotic-treated individuals and tissues.

Figure 4C. Total MP pg/mg fresh weight of whole individuals, dissected guts (gut) and rest-bodies (body) from L4 larval instars and newly hatched beetles (beetle p.h.) under *Sitotroga* eggs and *Sitotroga eggs*-antibiotic mix (ab). There exist significant differences between single pairs.

Figure 4C : Total MP (pg/mg fresh weight) of whole individuals, dissected guts (gut) and residual bodies (body) from larval instars and newly hatched beetles (beetle p.h.) under control diet (HS) and antibiotic mix (ab)

groups	1	2	3	4	5	6	7	8	9	10	11	12
	L4	L4 ab	L4 gut	L4 gut ab	L4 body	L4 body ab	beetle p.h.	beetle p.h. ab	beetle p.h. gut	beetle p.h. gut ab	beetle p.h. body	beetle p.h. body ab
	2,0967	2,5588	0	0,1696	0	0,2694	26,5055	3,1637	14,6209	14,8329	18,0047	6,3235
	3,2885	1,2851	0	1,3029	0,7029	0,5278	14,1237	11,615	38,7436	3,3282	20,0821	3,4434
	1,6655	0	1,974	1,5041	0,7761	0,3302	36,0807	7,4508	17,0013	5,023	25,1445	2,4013
	0,4972	0,8409	0	1,2354	1,2541	0	38,2384	7,5872	7,4877	2,9262	8,7155	6,0196
	2,0909	0,9221	16,6896	0	2,4137	0	28,9635	26,8293	11,0232	4,2768	8,9792	6,7995
							40,8307		9,7528		9,6255	
							30,2673					
							53,9689					
mean	1,92776	1,12138	3,73272	0,8424	1,02936	0,22548	33,6223375	11,3292	16,43825	6,07742	15,09191667	4,99746
Stdev	1,002752581	0,931243141	7,293377667	0,701187161	0,894036	0,226922392	11,69311127	9,165897698	11,44795858	4,962380025	6,961987846	1,949721112
stderr	0,448444587	0,416464593	3,26169765	0,313580431	0,399825	0,101482779	4,134139137	4,099114066	4,67360952	2,219243813	2,842219636	0,871941789
statistical analysis							**beeph**	**beephab**	**beetle p.h. gut**	**beetle p.h. gut ab**	**beephB**	**beephBab**
	not significant		not significant		not significant		**t-test**		**Mann-Whitney U**		**t-test**	
							**p=0.003**		**p=0.045**		**p=0.015**	

Figure 4. Influence of antibiotics on total MP content of *H. axyridis*.

A. Statistical analysis for Figure 4

Figure 4D. Total MP pg/sample of experiment presented in **C**. There exist significant differences between single pairs.

Figure 4D : Total MP (pg/sample) of whole individuals, dissected guts (gut) and residual bodies (body) from larval instars and newly hatched beetles (beetle p.h.) under control diet (HS) and antibiotic mix (ab)

groups	1	2	3	4	5	6	7	8	9	10	11	12
	L4	L4 ab	L4 gut	L4 gut ab	L4 body	L4 body ab	beetle p.h.	beetle p.h. ab	beetle p.h. gut	beetle p.h. gut ab	beetle p.h. body	beetle p.h. body ab
	28,3896	0,8225	0	0,0422	0	0,0977	332,3509	9,315	19,3397	9,7338	225,5781	62,4111
	63,0064	24,9981	0	1,9745	0,2121	2,5129	222,2952	218,5895	38,2826	4,5546	223,3982	19,527
	16,3506	0	0,0749	3,3691	0,1123	0,0802	1066,5227	16,8995	46,8977	14,6168	208,191	23,3688
	15,223	6,5312	0	0,0365	0,1526	0	358,5784	26,6053	9,6247	7,1489	8,3509	77,9453
	24,1368	13,7597	0,0804	0	0,0951	0	549,022	18,001	13,3699	9,9114	117,2094	41,3779
							985,6883		9,982		99,6285	
							73,5079					
							61,4088					
mean	29,42128	9,2223	0,03106	1,08446	0,11442	0,53816	456,171775	57,88206	22,9161	9,1931	147,05935	44,92602
stdev	19,55367107	10,39607307	0,042575087	1,530713126	0,078213	1,104826096	386,0719663	90,04713415	15,86895894	3,738959379	87,30479013	25,09190099
stderr	8,744667542	4,649265216	0,019040158	0,684555721	0,034978	0,494093251	136,4970527	40,27030263	6,478475358	1,672113467	35,64203132	11,22143926
statistical analysis	not significant		not significant		not significant		**beeph**	**beephab**	not significant		**beephB**	**beephBab**
							**Mann-Whitney U p=0.008**				**t-test p=0.034**	

Figure 4. Influence of antibiotics on total MP content of *H. axyridis*.

B. Data files for Figure 4

- **Total MP content: HS (honey syrup-*Sitotroga* eggs)**

HS (honey syrup-Sitotroga eggs) diet (feeding L1-L4)		total MP	average total MP	Stdev total MP	total MP	average total MP	Stdev total MP
Data File	stage	pg/mg FW	pg/mg FW	pg/mg FW	pg/sample	pg/sample	pg/sample
12_NTD_140407-005_HaL4_SH_1.D	L4	2,096670242	1,927745893	1,002757158	28,38959712	29,42127227	19,55368211
13_NTD_140407-005_HaL4_SH_2.D	L4	3,28847238			63,00641435		
15_NTD_140407-005_HaL4_SH_3.D	L4	1,665508168			16,35060299		
16_NTD_140407-005_HaL4_SH_4.D	L4	0,497158216			15,22298456		
17_NTD_140407-005_HaL4_SH_5.D	L4	2,090920461			24,13676232		
18_NTD_140407-005_HaL4_SH_gut_1.D	L4 gut	0	3,732710674	7,293363889	0	0,031063866	0,042579108
20_NTD_140407-005_HaL4_SH_gut_5.D	L4 gut	0			0		
22_NTD_140407-005_HaL4_SH_gut_9.D	L4 gut	1,973986257			0,074949173		
24_NTD_140407-005_HaL4_SH_gut_13.D	L4 gut	0			0		
26_NTD_140407-005_HaL4_SH_gut_16.D	L4 gut	16,68956711			0,080370154		
19_NTD_140407-005_HaL4_SH_rb_1.D	L4 residual body	0	1,029383295	0,894049891	0	0,114439428	0,078207201
21_NTD_140407-005_HaL4_SH_rb_5.D	L4 residual body	0,702931695			0,21207441		
23_NTD_140407-005_HaL4_SH_rb_9.D	L4 residual body	0,776130257			0,112330486		
25_NTD_140407-005_HaL4_SH_rb_13.D	L4 residual body	1,25410784			0,152646658		
28_NTD_140407-005_HaL4_SH_rb_16.D	L4 residual body	2,413746683			0,095145584		

HS (honey syrup-Sitotroga eggs) diet (feeding larvae to adults)		total MP	average total MP	Stdev total MP	total MP	average total MP	Stdev total MP
		pg/mg FW	pg/mg FW	pg/mg FW	pg/sample	pg/sample	pg/sample
65_NTD_140407-005_LHa_SH_1.D	larva to adult	26,50552334	33,62234578	11,69309556	332,3509185	456,1717709	386,0719625
66_NTD_140407-005_LHa_SH_2.D	larva to adult	14,12374203			222,2951904		
67_NTD_140407-005_LHa_SH_3.D	larva to adult	36,08073464			1066,522658		
68_NTD_140407-005_LHa_SH_4.D	larva to adult	38,2383795			358,5783635		
70_NTD_140407-005_LHa_SH_5.D	larva to adult	28,96349381			549,0220396		
06_NTD_140407-05_L_Ha_SH_1.D	larva to adult	40,83070053			985,6883086		
07_NTD_140407-05_L_Ha_SH_2.D	larva to adult	30,26730712			73,50786092		
08_NTD_140407-05_L_Ha_SH_3.D	larva to adult	53,96888525			61,40882815		
71_NTD_140407-005_LHa_SH_gut_1.D	larva to adult gut	14,62089821	16,43823414	11,44795664	19,33965067	22,91611515	15,86896553
73_NTD_140407-005_LHa_SH_gut_4.D	larva to adult gut	38,74358243			38,28264745		
75_NTD_140407-005_LHa_SH_gut_7.D	larva to adult gut	17,00126014			46,89771839		
77_NTD_140407-005_LHa_SH_gut_9.D	larva to adult gut	7,487670783			9,624740277		
79_NTD_140407-005_LHa_SH_gut_14.D	larva to adult gut	11,02319462			13,36994528		
10_NTD_140407-05_L_Ha_SH_3_gut.D	larva to adult gut	9,752798651			9,98198884		
72_NTD_140407-005_LHa_SH_rb_1.D	larva to adult rb	18,00466225	15,09191567	6,96200857	225,5781363	147,0593689	87,30480392
74_NTD_140407-005_LHa_SH_rb_4.D	larva to adult rb	20,08212467			223,3982195		
76_NTD_140407-005_LHa_SH_rb_7.D	larva to adult rb	25,14454712			208,1910215		
78_NTD_140407-005_LHa_SH_rb_9.D	larva to adult rb	8,715515018			8,350884943		
80_NTD_140407-005_LHa_SH_rb_14.D	larva to adult rb	8,979154592			117,2094401		
09_NTD_140407-05_L_Ha_SH_3_rb.D	larva to adult rb	9,625490348			99,62851108		

Figure 4. Influence of antibiotics on total MP content of *H. axyridis*.

B. Data files for Figure 4

- **Total MP content: HS (honey syrup-*Sitotroga* eggs)**

HS (honey syrup-Sitotroga eggs) diet		total MP	average total MP	Stdev total MP	total MP	average total MP	Stdev total MP
Data File	stage	pg/mg FW	pg/mg FW	pg/mg FW	pg/sample	pg/sample	pg/sample
21_NTD140407-05_160216_HSS-F10g.D	female gut	7,026077719	9,73494517	5,658094103	30,91474196	31,67766414	23,71922933
23_NTD140407-05_160216_HSS-F11g.D	female gut	8,806125716			11,97633097		
44_NTD140407-05_160216_HSS-F3g.D	female gut	8,146713093			23,7069351		
77_NTD140407-05_160216_HSS-F5g.D	female gut	16,8642328			60,03666878		
84_NTD140407-05_160216_HSS-F17g.D	female gut	17,99448879			68,1991125		
88_NTD140407-05_160216_HSS-F7g.D	female gut	6,96344876			21,23851872		
82_NTD140407-05_160216_HSS-F16g.D	female gut	2,343529308			5,671340926		
22_NTD140407-05_160216_HSS-F10b.D	female residual body	19,07091313	19,25125866	5,977344766	923,2229047	643,6887197	267,4495784
24_NTD140407-05_160216_HSS-F11b.D	female residual body	13,42909779			298,125971		
45_NTD140407-05_160216_HSS-F3b.D	female residual body	15,03873442			423,3403739		
78_NTD140407-05_160216_HSS-F5b.D	female residual body	30,75661731			955,3005335		
85_NTD140407-05_160216_HSS-F17b.D	female residual body	21,4717987			694,3979699		
87_NTD140407-05_160216_HSS-F6b.D	female residual body	20,59657354			806,3558542		
89_NTD140407-05_160216_HSS-F7b.D	female residual body	14,39507572			405,0774307		
16_NTD140407-05_160216_HSS-M18g.D	male gut	11,32501308	16,88674067	14,66062166	17,78027053	20,67521421	13,5439846
25_NTD140407-05_160216_HSS-M1g.D	male gut	44,12361504			44,56485119		
33_NTD140407-05_160216_HSS-M2g.D	male gut	18,49936474			16,83442191		
42_NTD140407-05_160216_HSS-M13g.D	male gut	17,63944626			27,16474724		
54_NTD140407-05_160216_HSS-M4g.D	male gut	7,074399868			7,923327852		
80_NTD140407-05_160216_HSS-M15g.D	male gut	2,65860504			9,783666548		
17_NTD140407-05_160216_HSS-M18b.D	male residual body	2,474163107	25,62961144	15,39791959	55,96556947	530,7803954	312,0100483
26_NTD140407-05_160216_HSS-M1b.D	male residual body	37,52370959			856,2910528		
34_NTD140407-05_160216_HSS-M2b.D	male residual body	32,43926381			623,4826505		
43_NTD140407-05_160216_HSS-M13b.D	male residual body	34,14352586			689,0163518		
55_NTD140407-05_160216_HSS-M4b.D	male residual body	9,801286486			238,5633131		
81_NTD140407-05_160216_HSS-M15b.D	male residual body	37,39571979			721,3634347		

HS (honey syrup-Sitotroga eggs) diet (feeding adults)		total MP	average total MP	Stdev total MP	total MP	average total MP	Stdev total MP
data file	stage	pg/mg FW	pg/mg FW	pg/mg FW	pg/sample	pg/sample	pg/sample
2_NTD_140407-05_SSH_1.D	adult	27,17024927	15,22276485	7,745463238	73,75073423	137,8257438	169,0470187
3_NTD_140407-05_SSH_2.D	adult	16,58353484			436,4497385		
4_NTD_140407-05_SSH_3.D	adult	14,30675417			26,16988175		
5_NTD_140407-05_SSH_4.D	adult	6,047398426			98,82121416		
7_NTD_140407-05_SSH_5.D	adult	12,00588754			53,9371502		

Figure 4. Influence of antibiotics on total MP content of *H. axyridis*.

B. Data files for Figure 4

- **Total MP content: HSAB (honey syrup-*Sitotroga* eggs-antibiotic mix) diet**

HSAB (honey syrup-Sitotroga eggs-antibiotics) diet, feeding L1-L4L		total MP	average total MP	Stdev total MP	total MP	average total MP	Stdev total MP
data file	stage	pg/mg FW	pg/mg FW	pg/mg FW	pg/sample	pg/sample	pg/sample
33_NTD_140407-005_HaL4_AB_1.D	L4	2,558824944	1,121384561	0,93125744	0,822494549	9,222316263	10,39609024
35_NTD_140407-005_HaL4_AB_2.D	L4	1,285143616			24,99814292		
36_NTD_140407-005_HaL4_AB_3.D	L4	0			0		
37_NTD_140407-005_HaL4_AB_4.D	L4	0,84088585			6,531228761		
38_NTD_140407-005_HaL4_AB_5.D	L4	0,922068394			13,75971508		
39_NTD_140407-005_HaL4_AB_gut_2.D	L4 gut	0,169643491	0,842387629	0,701158979	0,042158742	1,084452381	1,530729365
41_NTD_140407-005_HaL4_AB_gut_5.D	L4 gut	1,30286458			1,974472284		
43_NTD_140407-005_HaL4_AB_gut_8.D	L4 gut	1,504077699			3,369134045		
46_NTD_140407-005_HaL4_AB_gut_12.D	L4 gut	1,235352375			0,036496833		
47_NTD_140407-005_HaL4_AB_gut_16.D	L4 gut	0			0		
40_NTD_140407-005_HaL4_AB_rb_2.D	L4 residual body	0,269381368	0,22545893	0,226902008	0,097708579	0,538155043	1,104814834
42_NTD_140407-005_HaL4_AB_rb_5.D	L4 residual body	0,52775646			2,512874813		
44_NTD_140407-005_HaL4_AB_rb_8.D	L4 residual body	0,330156822			0,080191822		
46_NTD_140407-005_HaL4_AB_rb_12.D	L4 residual body	0			0		
48_NTD_140407-005_HaL4_AB_rb_16.D	L4 residual body	0			0		

HSAB (honey syrup-Sitotroga eggs-antibiotics) diet (feeding L1-L4)		total MP	average total MP	Stdev total MP	total MP	average total MP	Stdev total MP
		pg/mg FW	pg/mg FW	pg/mg FW	pg/sample	pg/sample	pg/sample
49_NTD_140407-005_LHa_AB_1.D	adult p.h.	3,163716901	11,32922738	9,165904363	9,315027724	57,88207427	90,04715045
50_NTD_140407-005_LHa_AB_2.D	adult p.h.	11,61504913			218,5895439		
51_NTD_140407-005_LHa_AB_3.D	adult p.h.	7,450835301			16,89948884		
52_NTD_140407-005_LHa_AB_4.D	adult p.h.	7,587202392			26,60531542		
53_NTD_140407-005_LHa_AB_5.D	adult p.h.	26,82933317			18,00099545		
54_NTD_140407-005_LHa_AB_gut_4.D	adult p.h. gut	14,83293478	6,077422336	4,962398088	9,73377527	9,193099099	3,738972792
57_NTD_140407-005_LHa_AB_gut_8.D	adult p.h. gut	3,328188086			4,554560296		
59_NTD_140407-005_LHa_AB_gut_10.D	adult p.h. gut	5,022964154			14,61682569		
61_NTD_140407-005_LHa_AB_gut_13.D	adult p.h. gut	2,926179371			7,14894855		
63_NTD_140407-005_LHa_AB_gut_16.D	adult p.h. gut	4,276845288			9,911385691		
55_NTD_140407-005_LHa_AB_rb_4.D	adult p.h. residual body	6,32346036	4,997436808	1,949726866	62,41112399	44,92602067	25,09192383
58_NTD_140407-005_LHa_AB_rb_8.D	adult p.h. residual body	3,443394335			19,52697628		
60_NTD_140407-005_LHa_AB_rb_10.D	adult p.h. residual body	2,40125244			23,36879092		
62_NTD_140407-005_LHa_AB_rb_13.D	adult p.h. residual body	6,019591302			77,94533058		
64_NTD_140407-005_LHa_AB_rb_16.D	adult p.h. residual body	6,799485603			41,37788158		

Figure 4. Influence of antibiotics on total MP content of *H. axyridis*.

B. Data files for Figure 4

- **Total MP content: vs. HSAB (honey syrup-*Sitotroga* eggs-antibiotic mix) diet**

HSAB (honey syrup-Sitotroga eggs-antibiotics) diet, male vs. female		total MP	average total MP	Stdev total MP	total MP	average total MP	Stdev total MP
data file	stage	pg/mg FW	pg/mg FW	pg/mg FW	pg/sample	pg/sample	pg/sample
02_NTD140407-05_150216_HSST-F1g.D	female gut	3,231582168	7,854576444	4,202734305	8,43442946	33,8016808	21,62506149
27_NTD140407-05_160216_HSST-F12g.D	female gut	2,133264107			9,98367602		
35_NTD140407-05_160216_HSST-F4g.D	female gut	11,14663777			44,92095023		
46_NTD140407-05_160216_HSST-F14g.D	female gut	13,32802239			69,43899666		
52_NTD140407-05_160216_HSST-F15g.D	female gut	6,156418513			16,19138069		
75_NTD140407-05_160216_HSST-F16g.D	female gut	12,77062687			46,8682006		
94_NTD140407-05_160216_HSST-F18g.D	female gut	6,620026909			46,34018836		
03_NTD140407-05_150216_HSST-F2b.D	female gut	7,450032822			28,2356244		
03_NTD140407-05_150216_HSST-F2b.D	female residual body	5,221212452	17,09347419	12,94284918	141,4426453	480,8534217	397,3677835
28_NTD140407-05_160216_HSST-F12b.D	female residual body	2,419282736			73,9090876		
36_NTD140407-05_160216_HSST-F4b.D	female residual body	33,91786958			1145,067277		
47_NTD140407-05_160216_HSST-F14b.D	female residual body	31,57047746			840,4061101		
53_NTD140407-05_160216_HSST-F15b.D	female residual body	6,611087085			162,8310749		
76_NTD140407-05_160216_HSST-F16b.D	female residual body	26,95856479			734,8904762		
95_NTD140407-05_160216_HSST-F18b.D	female residual body	7,678409354			193,0352112		
97_NTD140407-05_160216_HSST-M9b.D	female residual body	22,37089005			555,2454911		
06_NTD140407-05_160216_HSST-M10g.D	male gut	22,81404939	15,18143565	8,177087241	36,95876002	21,363991	16,85961986
08_NTD140407-05_160216_HSST-M19g.D	male gut	29,22435556			34,48473956		
12_NTD140407-05_160216_HSST-M2g.D	male gut	14,57503767			20,25930237		
14_NTD140407-05_160216_HSST-M11g.D	male gut	16,73182861			12,0469166		
19_NTD140407-05_160216_HSST-M20g.D	male gut	17,03278109			48,88408171		
31_NTD140407-05_160216_HSST-M3g.D	male gut	4,961739859			9,675392726		
37_NTD140407-05_160216_HSST-M13g.D	male gut	7,606537246			2,053765056		
90_NTD140407-05_160216_HSST-M8g.D	male gut	8,505155744			6,548969923		
07_NTD140407-05_160216_HSST-M10b.D	male residual body	38,72524818	22,87866957	13,11398246	858,9260046	499,0908804	291,9257256
09_NTD140407-05_160216_HSST-M19b.D	male residual body	36,32446125			779,886183		
13_NTD140407-05_160216_HSST-M2b.D	male residual body	10,97072478			257,0440816		
15_NTD140407-05_160216_HSST-M11b.D	male residual body	34,45798415			740,5020793		
32_NTD140407-05_160216_HSST-M3b.D	male residual body	13,70537234			350,8575319		
38_NTD140407-05_160216_HSST-M13b.D	male residual body	8,287919557			106,1682495		
91_NTD140407-05_160216_HSST-M8b.D	male residual body	17,67897672			400,252033		

HSAB (honey syrup-Sitotroga eggs-antibiotics) diet		total MP	average total MP	Stdev total MP	total MP	average total MP	Stdev total MP
data file	stage	pg/mg FW	pg/mg FW	pg/mg FW	pg/sample	pg/sample	pg/sample
05_NTD140407-06_TA_1.D	adult	14,47941317	14,26331481	6,19832569	614,0719124	609,6061429	293,0172562
07_NTD140407-06_TA_2.D	adult	24,12543548			1260,31275		
11_NTD140407-05_TA_4.D	adult	12,3582436			540,5495751		
13_NTD140407-05_TA-5.D	adult	12,74966681			588,0146333		
16_NTD140407-05_TA_6.D	adult	16,07906979			775,3327453		
18_NTD140407-05_TA_7.D	adult	5,678447453			221,8001575		
20_NTD140407-05_TA_8.D	adult	13,89869595			513,5568154		
22_NTD140407-05_TA_9.D	adult	5,575474978			244,9306158		
24_NTD140407-05_TA_10.D	adult	13,94718659			576,5766935		
26_NTD140407-05_TA_11.D	adult	23,74151424			760,9155313		

Data files for total MP content of diets (aphids and grapes) and tissues (fat body, muscle tissue, hemolymph) of adult beetles.

Measurements of MP content are presented only in pg/mg fresh weight, because tissues of individuals were pooled.

MP content of diets and tissues					
		total MP pg/mg FW		average total MP pg/mg FW	Stdev total MP pg/mg FW
**diets in Petri dish**					
60_NTD140407-05_160218_aphid1.D	aphid 1	0		0	0
61_NTD140407-05_160218_aphid2.D	aphid 2	0		0	0
62_NTD140407-05_16021_aphid3.D	aphid 3	0		0	0
63_NTD140407-05_16021_aphid4.D	aphid 4	0		0	0
69_NTD140407-05_grape1.D	grape 1	0		0	0
70_NTD140407-05_grape2.D	grape 2	0		0	0
71_NTD140407-05_grape3.D	grape 3	0		0	0
72_NTD140407-05_grape4.D	grape 4	0		0	0
03_NTD140407-05_honeysyrup_1.D	honeysyrup 1	0		0	0
04_NTD140407-05_honeysyrup_2.D	honeysyrup 2	0		0	0
05_NTD140407-05_honeysyrup_3.D	honeysyrup 3	0		0	0
06_NTD140407-05_honeysyrup_4.D	honeysyrup 4	0		0	0
07_NTD140407-05_honeysyrup_egg_1.D	honeysyrup-sitotroga 1	0		0	0
08_NTD140407-05_honeysyrup_egg_2.D	honeysyrup-sitotroga 2	0		0	0
09_NTD140407-05_honeysyrup_egg_3.D	honeysyrup-sitotroga 3	0		0	0
10_NTD140407-05_honeysyrup_egg_4.D	honeysyrup-sitotroga 4	0		0	0
**tissues after aphid diet in greenhouse**					
13_NTD140407-05_FB1.D	fat body 1	0		0	0
14_NTD140407-05_FB2.D	fat body 2	0		0	0
15_NTD140407-05_FB3.D	fat body 3	0		0	0
16_NTD140407-05_FB4.D	fat body 4	0		0	0
17_NTD140407-05_MUS1.D	muscle	0		0	0
18_NTD140407-05_MUS2.D	muscle	0		0	0
19_NTD140407-05_MUS3.D	muscle	0		0	0
			x 3 dilution		
65_NTD140407-05_haem1.D	hemolymph 1	13,49973228	40,49919684	37,65333515	1,65725429
66_NTD140407-05_haem2.D	hemolymph 2	10,77128528	32,31385584		
67_NTD140407-05_haem3.D	hemolymph 3	14,34989227	43,04967681		
68_NTD140407-05_haem4.D	hemolymph 4	11,58353704	34,75061112		
04_NTD140407-005_160121_F1g.D	female gut 1	9,462549574		9,065564209	7,509262066
08_NTD140407-005_160121_F2g.D	female gut 2	3,340969337			
21_NTD140407-005_160121_F5g.D	female gut 5	3,572895			
37_NTD140407-006_160121_F6g.D	female gut 6-11 pooled/6 (Proben 6-11)	2,543916023			
06_NTD140407-005_160121_M1g.D	male gut 1	24,01613391			
10_NTD140407-005_160121_M2g.D	male gut 2	3,199143751			
15_NTD140407-005_160121_M3g.D	male gut 3	17,91399861			
19_NTD140407-005_160121_M4g.D	male gut 4	15,24693243			
23_NTD140407-005_160121_M5g.D	male gut 5	4,179123769			
38_NTD140407-006_160121_M6-10g.D	male gut 6-11 pooled /6 (Proben 6-11)	7,179979686			

Data files for SBMP, IPMP, and IBMP content of adult *H. axyridis* feeding on HS and HSAB diet.

- **SBMP**

HS (honey syrup-Sitotroga eggs diet)		SBMP pg/mg FW	average SBMP pg/mg FW	Stdev SBMP pg/mg FW	SBMP pg/sample	average SBMP pg/sample	Stdev SBMP pg/sample
21_NTD140407-05_160216_HSS-F10g.D	female gut	2,395632573	1,78548577	2,529819264	10,54078332	6,044357188	7,925239082
23_NTD140407-05_160216_HSS-F11g.D	female gut	0			0		
44_NTD140407-05_160216_HSS-F3g.D	female gut	6,455594521			18,78578006		
77_NTD140407-05_160216_HSS-F5g.D	female gut	3,647173297			12,98393694		
84_NTD140407-05_160216_HSS-F17g.D	female gut	0			0		
88_NTD140407-05_160216_HSS-F7g.D	female gut	0			0		
82_NTD140407-05_160216_HSS-F6g.D	female gut	0			0		
22_NTD140407-05_160216_HSS-F10b.D	female residual body	5,239207204	4,100338025	3,854626754	253,6300207	135,8599594	119,6448485
24_NTD140407-05_160216_HSS-F11b.D	female residual body	0,33978816			7,543297157		
45_NTD140407-05_160216_HSS-F3b.D	female residual body	11,61645386			327,0031761		
78_NTD140407-05_160216_HSS-F5b.D	female residual body	4,902624445			152,2755153		
85_NTD140407-05_160216_HSS-F17b.D	female residual body	4,0435681			130,7689923		
87_NTD140407-05_160216_HSS-F6b.D	female residual body	0,702990838			27,52209131		
89_NTD140407-05_160216_HSS-F7b.D	female residual body	1,857733571			52,2766227		
16_NTD140407-05_160216_HSS-M18g.D	male gut	0	6,688459236	6,623492139	0	8,171170454	6,180257349
25_NTD140407-05_160216_HSS-M1g.D	male gut	18,96251785			19,15214303		
33_NTD140407-05_160216_HSS-M2g.D	male gut	7,080383622			6,443149096		
42_NTD140407-05_160216_HSS-M13g.D	male gut	4,813601986			7,412947058		
54_NTD140407-05_160216_HSS-M4g.D	male gut	2,19985209			8,09545569		
80_NTD140407-05_160216_HSS-M15g.D	male gut	7,074399868			7,923327852		
17_NTD140407-05_160216_HSS-M18b.D	male residual body	0	10,23148142	6,705438727	0	213,5866166	135,1851105
26_NTD140407-05_160216_HSS-M1b.D	male residual body	14,9346881			340,8095824		
34_NTD140407-05_160216_HSS-M2b.D	male residual body	11,53380063			221,6796481		
43_NTD140407-05_160216_HSS-M13b.D	male residual body	7,924340967			159,9132007		
55_NTD140407-05_160216_HSS-M4b.D	male residual body	7,596691721			184,9034765		
81_NTD140407-05_160216_HSS-M15b.D	male residual body	19,39936713			374,2137919		

HSAB (honey syrup-Sitotroga eggs-antibiotics diet) data file		SBMP pg/mg FW	average SBMP pg/mg FW	Stdev SBMP pg/mg FW	SBMP pg/sample	average SBMP pg/sample	Stdev SBMP pg/sample
02_NTD140407-05_150216_HSST-F1g.D	female gut	0	1,263823255	1,965202345	0	6,720302829	9,01850465
27_NTD140407-05_160216_HSST-F12g.D	female gut	0			0		
35_NTD140407-05_160216_HSST-F4g.D	female gut	0			0		
46_NTD140407-05_160216_HSST-F14g.D	female gut	4,79598985			24,98710712		
52_NTD140407-05_160216_HSST-F15g.D	female gut	3,816640704			10,03776505		
75_NTD140407-05_160216_HSST-F16g.D	female gut	1,497955489			5,497496644		
94_NTD140407-05_160216_HSST-F18g.D	female gut	0			13,24005382		
96_NTD140407-05_160216_HSST-M9g/F.D	female gut	0			0		
03_NTD140407-05_150216_HSST-F2b.D	female residual body	0	2,338486599	3,363438018	0	61,32950516	88,64593584
28_NTD140407-05_160216_HSST-F12b.D	female residual body	0,249559156			7,624032223		
36_NTD140407-05_160216_HSST-F4b.D	female residual body	0,407610407			13,76092735		
47_NTD140407-05_160216_HSST-F14b.D	female residual body	9,914907855			263,9348471		
53_NTD140407-05_160216_HSST-F15b.D	female residual body	4,431186964			109,1401349		
76_NTD140407-05_160216_HSST-F16b.D	female residual body	1,583964545			43,17887351		
95_NTD140407-05_160216_HSST-F18b.D	female residual body	1,132341211			28,46705806		
97_NTD140407-05_160216_HSST-M9b/F.D	female residual body	0,988322649			24,53016815		
06_NTD140407-05_160216_HSST-M10g.D	male gut	10,1418166	3,586602911	4,328399445	16,42974289	6,331780539	7,900898901
08_NTD140407-05_160216_HSST-M19g.D	male gut	3,318221832			3,915501762		
12_NTD140407-05_160216_HSST-M2g.D	male gut	9,060197901			12,59367508		
14_NTD140407-05_160216_HSST-M11g.D	male gut	0			0		
19_NTD140407-05_160216_HSST-M20g.D	male gut	6,17258696			17,71532458		
31_NTD140407-05_160216_HSST-M3g.D	male gut	0			0		
37_NTD140407-05_160216_HSST-M13g.D	male gut	0			0		
90_NTD140407-05_160216_HSST-M8g.D	male gut	0			0		
07_NTD140407-05_160216_HSST-M10b.D	male residual body	13,7393151	4,020268897	4,825171935	304,7380089	91,0615949	107,6848653
09_NTD140407-05_160216_HSST-M19b.D	male residual body	2,3875661			51,26104418		
13_NTD140407-05_160216_HSST-M2b.D	male residual body	7,040806155			164,9660882		
15_NTD140407-05_160216_HSST-M11b.D	male residual body	1,879736606			40,39553966		
32_NTD140407-05_160216_HSST-M3b.D	male residual body	2,031063195			51,99521779		
38_NTD140407-05_160216_HSST-M13b.D	male residual body	0			0		
91_NTD140407-05_160216_HSST-M8b.D	male residual body	1,06339512			24,07526551		

Data files for SBMP, IPMP, and IBMP content of adult *H. axyridis* feeding on HS and HSAB diet.

- **IPMP**

HS (honey syrup-Sitotroga eggs diet) data file		IPMP pg/mg FW	average IPMP pg/mg FW	Stdev IPMP pg/mg FW	IPMP pg/sample	average IPMP pg/sample	Stdev IPMP pg/sample
21_NTD140407-05_160216_HSS-F10g.D	female gut	4,630445145	7,949459399	5,943700561	20,37395864	25,63330695	23,56409978
23_NTD140407-05_160216_HSS-F11g.D	female gut	8,806125716			11,97633097		
44_NTD140407-05_160216_HSS-F3g.D	female gut	1,691118573			4,921155047		
77_NTD140407-05_160216_HSS-F5g.D	female gut	13,21705951			47,05273184		
84_NTD140407-05_160216_HSS-F17g.D	female gut	17,99448879			68,1991125		
88_NTD140407-05_160216_HSS-F7g.D	female gut	6,96344876			21,23851872		
82_NTD140407-05_160216_HSS-F6g.D	female gut	2,343529308			5,671340926		
22_NTD140407-05_160216_HSS-F10b.D	female residual body	13,83170593	15,15092063	6,983517465	669,592884	507,8287603	267,8372243
24_NTD140407-05_160216_HSS-F11b.D	female residual body	13,08930963			290,5826738		
45_NTD140407-05_160216_HSS-F3b.D	female residual body	3,422280561			96,33719779		
78_NTD140407-05_160216_HSS-F5b.D	female residual body	25,85399286			803,0250182		
85_NTD140407-05_160216_HSS-F17b.D	female residual body	17,4282306			563,6289775		
87_NTD140407-05_160216_HSS-F6b.D	female residual body	19,8935827			778,8337629		
89_NTD140407-05_160216_HSS-F7b.D	female residual body	12,53734215			352,800808		
16_NTD140407-05_160216_HSS-M18g.D	male gut	11,32501308	10,19828143	9,299601421	17,78027053	12,50404376	10,24499206
25_NTD140407-05_160216_HSS-M1g.D	male gut	25,16109719			25,41270816		
33_NTD140407-05_160216_HSS-M2g.D	male gut	11,41898111			10,39127281		
42_NTD140407-05_160216_HSS-M13g.D	male gut	12,82584427			19,75180018		
54_NTD140407-05_160216_HSS-M4g.D	male gut	0,45875295			1,688210858		
80_NTD140407-05_160216_HSS-M15g.D	male gut	0			0		
17_NTD140407-05_160216_HSS-M18b.D	male residual body	2,474163107	15,39813002	10,45929177	55,96556947	317,1937788	214,4621091
26_NTD140407-05_160216_HSS-M1b.D	male residual body	22,58902149			515,4814704		
34_NTD140407-05_160216_HSS-M2b.D	male residual body	20,90546318			401,8030024		
43_NTD140407-05_160216_HSS-M13b.D	male residual body	26,21918489			529,1031511		
55_NTD140407-05_160216_HSS-M4b.D	male residual body	2,204594764			53,65983657		
81_NTD140407-05_160216_HSS-M15b.D	male residual body	17,99635266			347,1496428		

HSAB (honey syrup-Sitotroga eggs-antibiotics diet) data file		IPMP pg/mg FW	average IPMP pg/mg FW	Stdev IPMP pg/mg FW	IPMP pg/sample	average IPMP pg/sample	Stdev IPMP pg/sample
02_NTD140407-05_150216_HSST-F1g.D	female gut	3,231582168	6,590753188	3,711146468	8,43442946	23,77136452	17,86669225
27_NTD140407-05_160216_HSST-F12g.D	female gut	2,133264107			9,98367602		
35_NTD140407-05_160216_HSST-F4g.D	female gut	11,14663777			44,92095023		
46_NTD140407-05_160216_HSST-F14g.D	female gut	8,532032541			44,45188954		
52_NTD140407-05_160216_HSST-F15g.D	female gut	2,339777809			6,153615638		
75_NTD140407-05_160216_HSST-F16g.D	female gut	11,27267138			41,37070395		
94_NTD140407-05_160216_HSST-F18g.D	female gut	6,620026909			6,620026909		
96_NTD140407-05_160216_HSST-M9g/F.D	female gut	7,450032822			28,2356244		
03_NTD140407-05_150216_HSST-F2b.D	female residual body	5,221212452	14,75498759	12,1354272	141,4426453	419,5239165	381,4744817
28_NTD140407-05_160216_HSST-F12b.D	female residual body	2,16972358			66,28505537		
36_NTD140407-05_160216_HSST-F4b.D	female residual body	33,51025917			1131,30635		
47_NTD140407-05_160216_HSST-F14b.D	female residual body	21,65556961			576,471263		
53_NTD140407-05_160216_HSST-F15b.D	female residual body	2,17990012			53,69093997		
76_NTD140407-05_160216_HSST-F16b.D	female residual body	25,37460025			691,7116027		
95_NTD140407-05_160216_HSST-F18b.D	female residual body	6,546068143			164,5681531		
97_NTD140407-05_160216_HSST-M9b/F.D	female residual body	21,3825674			530,7153229		
06_NTD140407-05_160216_HSST-M10g.D	male gut	12,67223279	11,59483273	6,962341435	20,52901713	15,03221046	11,11165205
08_NTD140407-05_160216_HSST-M19g.D	male gut	25,90613373			30,5692378		
12_NTD140407-05_160216_HSST-M2g.D	male gut	5,514839773			7,665627285		
14_NTD140407-05_160216_HSST-M11g.D	male gut	16,73182861			12,0469166		
19_NTD140407-05_160216_HSST-M20g.D	male gut	10,86019412			31,16875714		
31_NTD140407-05_160216_HSST-M3g.D	male gut	4,961739859			9,675392726		
37_NTD140407-05_160216_HSST-M13g.D	male gut	7,606537246			2,053765056		
90_NTD140407-05_160216_HSST-M8g.D	male gut	8,505155744			6,548969923		
07_NTD140407-05_160216_HSST-M10b.D	male residual body	24,98593308	18,85840067	11,86295286	554,1879957	408,0292855	262,4660317
09_NTD140407-05_160216_HSST-M19b.D	male residual body	33,93689515			728,6251389		
13_NTD140407-05_160216_HSST-M2b.D	male residual body	3,929918625			92,07799338		
15_NTD140407-05_160216_HSST-M11b.D	male residual body	32,57824754			700,1065397		
32_NTD140407-05_160216_HSST-M3b.D	male residual body	11,67430915			298,8623141		
38_NTD140407-05_160216_HSST-M13b.D	male residual body	8,287919557			106,1682495		
91_NTD140407-05_160216_HSST-M8b.D	male residual body	16,6155816			376,1767675		

Data files for SBMP, IPMP, and IBMP content of adult *H. axyridis* feeding on HS and HSAB diet.

- **IBMP**

HS (honey syrup-Sitotroga eggs diet) data file		IBMP pg/mg FW	average IBMP pg/mg FW	Stdev IBMP pg/mg FW	IBMP pg/sample	average IBMP pg/sample	Stdev IBMP pg/sample
21_NTD140407-05_160216_HSS-F10g.D	female gut	0	0	0	0	0	0
23_NTD140407-05_160216_HSS-F11g.D	female gut	0			0		
44_NTD140407-05_160216_HSS-F3g.D	female gut	0			0		
77_NTD140407-05_160216_HSS-F5g.D	female gut	0			0		
84_NTD140407-05_160216_HSS-F17g.D	female gut	0			0		
88_NTD140407-05_160216_HSS-F7g.D	female gut	0			0		
82_NTD140407-05_160216_HSS-F6g.D	female gut	0			0		
22_NTD140407-05_160216_HSS-F10b.D	female residual body	0	0	0	0	0	0
24_NTD140407-05_160216_HSS-F11b.D	female residual body	0			0		
45_NTD140407-05_160216_HSS-F3b.D	female residual body	0			0		
78_NTD140407-05_160216_HSS-F5b.D	female residual body	0			0		
85_NTD140407-05_160216_HSS-F17b.D	female residual body	0			0		
87_NTD140407-05_160216_HSS-F6b.D	female residual body	0			0		
89_NTD140407-05_160216_HSS-F7b.D	female residual body	0			0		
16_NTD140407-05_160216_HSS-M18g.D	male gut	0	0	0	0	0	0
25_NTD140407-05_160216_HSS-M1g.D	male gut	0			0		
33_NTD140407-05_160216_HSS-M2g.D	male gut	0			0		
42_NTD140407-05_160216_HSS-M13g.D	male gut	0			0		
54_NTD140407-05_160216_HSS-M4g.D	male gut	0			0		
80_NTD140407-05_160216_HSS-M15g.D	male gut	0			0		
17_NTD140407-05_160216_HSS-M18b.D	male residual body	0	0	0	0	0	0
26_NTD140407-05_160216_HSS-M1b.D	male residual body	0			0		
34_NTD140407-05_160216_HSS-M2b.D	male residual body	0			0		
43_NTD140407-05_160216_HSS-M13b.D	male residual body	0			0		
55_NTD140407-05_160216_HSS-M4b.D	male residual body	0			0		
81_NTD140407-05_160216_HSS-M15b.D	male residual body	0			0		

HSAB (honey syrup-Sitotroga eggs-antibiotics diet) data file		IBMP pg/mg FW	average IBMP pg/mg FW	Stdev IBMP pg/mg FW	IBMP pg/sample	average IBMP pg/sample	Stdev IBMP pg/sample
02_NTD140407-05_150216_HSST-F1g.D	female gut	0	0	0	0	0	0
27_NTD140407-05_160216_HSST-F12g.D	female gut	0			0		
35_NTD140407-05_160216_HSST-F4g.D	female gut	0			0		
46_NTD140407-05_160216_HSST-F14g.D	female gut	0			0		
52_NTD140407-05_160216_HSST-F15g.D	female gut	0			0		
75_NTD140407-05_160216_HSST-F16g.D	female gut	0			0		
94_NTD140407-05_160216_HSST-F18g.D	female gut	0			0		
96_NTD140407-05_160216_HSST-M9g/F.D	female gut	0			0		
03_NTD140407-05_150216_HSST-F2b.D	female residual body	0	0	0	0	0	0
28_NTD140407-05_160216_HSST-F12b.D	female residual body	0			0		
36_NTD140407-05_160216_HSST-F4b.D	female residual body	0			0		
47_NTD140407-05_160216_HSST-F14b.D	female residual body	0			0		
53_NTD140407-05_160216_HSST-F15b.D	female residual body	0			0		
76_NTD140407-05_160216_HSST-F16b.D	female residual body	0			0		
95_NTD140407-05_160216_HSST-F18b.D	female residual body	0			0		
97_NTD140407-05_160216_HSST-M9b/F.D	female residual body	0			0		
06_NTD140407-05_160216_HSST-M10g.D	male gut	0	0	0	0	0	0
08_NTD140407-05_160216_HSST-M19g.D	male gut	0			0		
12_NTD140407-05_160216_HSST-M2g.D	male gut	0			0		
14_NTD140407-05_160216_HSST-M11g.D	male gut	0			0		
19_NTD140407-05_160216_HSST-M20g.D	male gut	0			0		
31_NTD140407-05_160216_HSST-M3g.D	male gut	0			0		
37_NTD140407-05_160216_HSST-M13g.D	male gut	0			0		
90_NTD140407-05_160216_HSST-M8g.D	male gut	0			0		
07_NTD140407-05_160216_HSST-M10b.D	male residual body	0	0	0	0	0	0
09_NTD140407-05_160216_HSST-M19b.D	male residual body	0			0		
13_NTD140407-05_160216_HSST-M2b.D	male residual body	0			0		
15_NTD140407-05_160216_HSST-M11b.D	male residual body	0			0		
32_NTD140407-05_160216_HSST-M3b.D	male residual body	0			0		
38_NTD140407-05_160216_HSST-M13b.D	male residual body	0			0		
91_NTD140407-05_160216_HSST-M8b.D	male residual body	0			0		

Data files for SBMP, IPMP, and IBMP content during development of *H. axyridis* feeding on HS and HSAB diet

- **SBMP**

HS (honey syrup-Sitotroga eggs diet) (feeding L1-L4)		SBMP	average SBMP	Stdev SBMP	SBMP	average SBMP	Stdev SBMP
data file		pg/mg FW	pg/mg FW	pg/mg FW	pg/sample	pg/sample	pg/sample
12_NTD_140407-005_HaL4_SH_1.D	L4	0,516901616	0,843146222	0,624244316	16,61838695	25,70702226	17,56191424
13_NTD_140407-005_HaL4_SH_2.D	L4	1,939640142			56,48232094		
15_NTD_140407-005_HaL4_SH_3.D	L4	0,489095038			16,14991815		
16_NTD_140407-005_HaL4_SH_4.D	L4	0,497158216			15,22298456		
17_NTD_140407-005_HaL4_SH_5.D	L4	0,772936096			24,06150068		
18_NTD_140407-005_HaL4_SH_gut_1.D	L4 gut	0	0	0	0	0	0
20_NTD_140407-005_HaL4_SH_gut_5.D	L4 gut	0			0		
22_NTD_140407-005_HaL4_SH_gut_9.D	L4 gut	0			0		
24_NTD_140407-005_HaL4_SH_gut_13.D	L4 gut	0			0		
26_NTD_140407-005_HaL4_SH_gut_16.D	L4 gut	0			0		
19_NTD_140407-005_HaL4_SH_rb_1.D	L4 residual body	0	0	0	0	0	0
21_NTD_140407-005_HaL4_SH_rb_5.D	L4 residual body	0			0		
23_NTD_140407-005_HaL4_SH_rb_9.D	L4 residual body	0			0		
25_NTD_140407-005_HaL4_SH_rb_13.D	L4 residual body	0			0		
28_NTD_140407-005_HaL4_SH_rb_16.D	L4 residual body	0			0		

HSAB (honey syrup-Sitotroga eggs-antibiotics diet) (feeding L1-L4)		SBMP	average SBMP	Stdev SBMP	SBMP	average SBMP	Stdev SBMP
data file	Data File	pg/mg FW	pg/mg FW	pg/mg FW	pg/sample	pg/sample	pg/sample
33_NTD_140407-005_HaL4_AB_1.D	L4	0	0,275388121	0,295836654	0	8,997183478	10,506398
35_NTD_140407-005_HaL4_AB_2.D	L4	0,700421308			24,85094802		
36_NTD_140407-005_HaL4_AB_3.D	L4	0			0		
37_NTD_140407-005_HaL4_AB_4.D	L4	0,268112655			6,461514979		
38_NTD_140407-005_HaL4_AB_5.D	L4	0,408406642			13,67345439		
39_NTD_140407-005_HaL4_AB_gut_2.D	L4 gut	0	0,411080868	0,655986545	0	1,061960764	1,53935539
41_NTD_140407-005_HaL4_AB_gut_5.D	L4 gut	0,551326641			1,940669778		
43_NTD_140407-005_HaL4_AB_gut_8.D	L4 gut	1,504077699			3,369134045		
46_NTD_140407-005_HaL4_AB_gut_12.D	L4 gut	0			0		
47_NTD_140407-005_HaL4_AB_gut_16.D	L4 gut	0			0		
40_NTD_140407-005_HaL4_AB_rb_2.D	L4 residual body	0	0,033458845	0,074816253	0	0,48247655	1,078850363
42_NTD_140407-005_HaL4_AB_rb_5.D	L4 residual body	0,167294227			2,412382748		
44_NTD_140407-005_HaL4_AB_rb_8.D	L4 residual body	0			0		
46_NTD_140407-005_HaL4_AB_rb_12.D	L4 residual body	0			0		
48_NTD_140407-005_HaL4_AB_rb_16.D	L4 residual body	0			0		

Data files for SBMP, IPMP, and IBMP content during development of *H. axyridis* feeding on HS and HSAB diet

- **SBMP**

HS (honey syrup-Sitotroga eggs diet) (feeding larvae to adults) data file		SBMP pg/mg FW	average SBMP pg/mg FW	Stdev SBMP pg/mg FW	SBMP pg/sample	average SBMP pg/sample	Stdev SBMP pg/sample
65_NTD_140407-005_LHa_SH_1.D	adult	11,67112517	15,39207675	12,32109854	332,1602223	438,9597135	370,8639809
66_NTD_140407-005_LHa_SH_2.D	adult	8,356663206			222,2036746		
67_NTD_140407-005_LHa_SH_3.D	adult	32,90550708			1066,138429		
68_NTD_140407-005_LHa_SH_4.D	adult	15,89736892			358,4856691		
70_NTD_140407-005_LHa_SH_5.D	adult	17,92422977			532,5288664		
06_NTD_140407-05_L_Ha_SH_1.D	adult	33,00710441			888,5512506		
07_NTD_140407-05_L_Ha_SH_2.D	adult	1,741207547			50,37313433		
08_NTD_140407-05_L_Ha_SH_3.D	adult	1,633407873			61,23646115		
71_NTD_140407-005_LHa_SH_gut_1.D	adult gut	9,417461588	11,01941109	11,32040486	19,30579626	22,65909814	15,46583132
73_NTD_140407-005_LHa_SH_gut_4.D	adult gut	33,55082274			38,24793792		
75_NTD_140407-005_LHa_SH_gut_7.D	adult gut	9,289688305			45,5194727		
77_NTD_140407-005_LHa_SH_gut_9.D	adult gut	3,789780856			9,588145565		
79_NTD_140407-005_LHa_SH_gut_14.D	adult gut	6,380801473			13,33587508		
10_NTD_140407-05_L_Ha_SH_3_gut.D	adult gut	3,687911592			9,957361297		
72_NTD_140407-005_LHa_SH_rb_1.D	adult residual body	11,41633414	6,626115613	4,477404438	221,1343922	122,8195331	95,80162286
74_NTD_140407-005_LHa_SH_rb_4.D	adult residual body	11,54760566			208,6652343		
76_NTD_140407-005_LHa_SH_rb_7.D	adult residual body	8,088442243			176,4898097		
78_NTD_140407-005_LHa_SH_rb_9.D	adult residual body	0,460934151			8,241502612		
80_NTD_140407-005_LHa_SH_rb_14.D	adult residual body	4,555465897			112,4288983		
09_NTD_140407-05_L_Ha_SH_3_rb.D	adult residual body	3,687911592			9,957361297		

HSAB (honey syrup-Sitotroga eggs-antibiotics diet)(feeding larvae to adult) data file		SBMP pg/mg FW	average SBMP pg/mg FW	Stdev SBMP pg/mg FW	SBMP pg/sample	average SBMP pg/sample	Stdev SBMP pg/sample
49_NTD_140407-005_LHa_AB_1.D	adult	0,389192301	2,031086577	3,092992343	9,239425225	55,39232075	84,71488816
50_NTD_140407-005_LHa_AB_2.D	adult	7,554452196			206,538723		
51_NTD_140407-005_LHa_AB_3.D	adult	0,602713997			16,81572051		
52_NTD_140407-005_LHa_AB_4.D	adult	0,887984705			26,50634345		
53_NTD_140407-005_LHa_AB_5.D	adult	0,721089686			17,86139152		
54_NTD_140407-005_LHa_AB_gut_4.D	adult gut	3,644386851	3,511104352	1,061534139	9,694069023	9,158835694	3,756714356
57_NTD_140407-005_LHa_AB_gut_8.D	adult gut	2,337535576			4,511443662		
59_NTD_140407-005_LHa_AB_gut_10.D	adult gut	5,022964154			14,61682569		
61_NTD_140407-005_LHa_AB_gut_13.D	adult gut	2,677203822			7,094590127		
63_NTD_140407-005_LHa_AB_gut_16.D	adult gut	3,87343136			9,877249968		
55_NTD_140407-005_LHa_AB_rb_4.D	adult residual body	3,384883828	2,284923461	1,143048324	62,31571127	44,81800756	25,10022702
58_NTD_140407-005_LHa_AB_rb_8.D	adult residual body	1,063743257			19,4345893		
60_NTD_140407-005_LHa_AB_rb_10.D	adult residual body	1,285910419			23,21068306		
62_NTD_140407-005_LHa_AB_rb_13.D	adult residual body	3,515694834			77,83748362		
64_NTD_140407-005_LHa_AB_rb_16.D	adult residual body	2,174384969			41,29157057		

Data files for SBMP, IPMP, and IBMP content during development of *H. axyridis* feeding on HS and HSAB diet

- **IPMP**

HS (honey syrup-Sitotroga eggs diet) (feeding L1-L4)		IPMP	average IPMP	Stdev IPMP	IPMP	average IPMP	Stdev IPMP
data set		pg/mg FW	pg/mg FW	pg/mg FW	pg/sample	pg/sample	pg/sample
12_NTD_140407-005_HaL4_SH_1.D	L4	1,216874656	0,967941871	0,545579454	0,104169032	0,097261119	0,072054864
13_NTD_140407-005_HaL4_SH_2.D	L4	1,128437206			0,106190081		
15_NTD_140407-005_HaL4_SH_3.D	L4	1,17641313			0,200684839		
16_NTD_140407-005_HaL4_SH_4.D	L4	0			0		
17_NTD_140407-005_HaL4_SH_5.D	L4	1,317984364			0,075261642		
18_NTD_140407-005_HaL4_SH_gut_1.D	L4 gut	0	3,732710674	7,293363889	0	0,031063866	0,042579108
20_NTD_140407-005_HaL4_SH_gut_5.D	L4 gut	0			0		
22_NTD_140407-005_HaL4_SH_gut_9.D	L4 gut	1,973986257			0,074949173		
24_NTD_140407-005_HaL4_SH_gut_13.D	L4 gut	0			0		
26_NTD_140407-005_HaL4_SH_gut_16.D	L4 gut	16,68956711			0,080370154		
19_NTD_140407-005_HaL4_SH_rb_1.D	L4 residual body	0	1,029383295	0,894049891	0	0,114439428	0,078207201
21_NTD_140407-005_HaL4_SH_rb_5.D	L4 residual body	0,702931695			0,21207441		
23_NTD_140407-005_HaL4_SH_rb_9.D	L4 residual body	0,776130257			0,112330486		
25_NTD_140407-005_HaL4_SH_rb_13.D	L4 residual body	1,25410784			0,152646658		
28_NTD_140407-005_HaL4_SH_rb_16.D	L4 residual body	2,413746683			0,095145584		

HSAB (honey syrup-Sitotroga eggs-antibiotics diet) (feeding L1-L4)		IPMP	average IPMP	Stdev IPMP	IPMP	average IPMP	Stdev IPMP
data file		pg/mg FW	pg/mg FW	pg/mg FW	pg/sample	pg/sample	pg/sample
33_NTD_140407-005_HaL4_AB_1.D	L4	2,558824944	0,84599644	0,987781975	0,822494549	0,225132785	0,338023922
35_NTD_140407-005_HaL4_AB_2.D	L4	0,584722308			0,147194897		
36_NTD_140407-005_HaL4_AB_3.D	L4	0			0		
37_NTD_140407-005_HaL4_AB_4.D	L4	0,572773195			0,069713782		
38_NTD_140407-005_HaL4_AB_5.D	L4	0,513661752			0,086260697		
39_NTD_140407-005_HaL4_AB_gut_2.D	L4 gut	0,169643491	0,431306761	0,545333858	0,042158742	0,022491616	0,020752251
41_NTD_140407-005_HaL4_AB_gut_5.D	L4 gut	0,751537939			0,033802506		
43_NTD_140407-005_HaL4_AB_gut_8.D	L4 gut	0			0		
46_NTD_140407-005_HaL4_AB_gut_12.D	L4 gut	1,235352375			0,036496833		
47_NTD_140407-005_HaL4_AB_gut_16.D	L4 gut	0			0		
40_NTD_140407-005_HaL4_AB_rb_2.D	L4 residual body	0,269381368	0,192000085	0,178313423	0,097708579	0,055678493	0,051419541
42_NTD_140407-005_HaL4_AB_rb_5.D	L4 residual body	0,360462234			0,100492065		
44_NTD_140407-005_HaL4_AB_rb_8.D	L4 residual body	0,330156822			0,080191822		
46_NTD_140407-005_HaL4_AB_rb_12.D	L4 residual body	0			0		
48_NTD_140407-005_HaL4_AB_rb_16.D	L4 residual body	0			0		

Data files for SBMP, IPMP, and IBMP content during development of *H. axyridis* feeding on HS and HSAB diet

- **IPMP**

HS (honey syrup-Sitotroga eggs diet (feeding larvae to adults)) data file		IPMP pg/mg FW	average IPMP pg/mg FW	Stdev IPMP pg/mg FW	IPMP pg/sample	average IPMP pg/sample	Stdev IPMP pg/sample
65_NTD_140407-005_LHa_SH_1.D	adult	14,83439817	17,61188281	16,54927874	0,190696231	0,173885177	0,107898295
66_NTD_140407-005_LHa_SH_2.D	adult	5,767078828			0,091515715		
67_NTD_140407-005_LHa_SH_3.D	adult	3,175227569			0,384228246		
68_NTD_140407-005_LHa_SH_4.D	adult	22,34101058			0,092694363		
70_NTD_140407-005_LHa_SH_5.D	adult	10,48678748			0,079094618		
06_NTD_140407-05_L_Ha_SH_1.D	adult	4,219187329			0,106373343		
07_NTD_140407-05_L_Ha_SH_2.D	adult	27,73589513			0,274111904		
08_NTD_140407-05_L_Ha_SH_3.D	adult	52,33547738			0,172366999		
71_NTD_140407-005_LHa_SH_gut_1.D	adult gut	5,203436619	5,376769439	1,283127485	0,033854412	0,050954334	0,044735354
73_NTD_140407-005_LHa_SH_gut_4.D	adult gut	5,192759696			0,034709528		
75_NTD_140407-005_LHa_SH_gut_7.D	adult gut	7,459250184			0,141869603		
77_NTD_140407-005_LHa_SH_gut_9.D	adult gut	3,697889927			0,036594711		
79_NTD_140407-005_LHa_SH_gut_14.D	adult gut	4,642393148			0,034070204		
10_NTD_140407-05_L_Ha_SH_3_gut.D	adult gut	6,064887059			0,024627543		
72_NTD_140407-005_LHa_SH_rb_1.D	adult residual body	6,363850872	7,935481193	4,036606524	0,095619993	0,100915334	0,009205398
74_NTD_140407-005_LHa_SH_rb_4.D	adult residual body	7,725152874			0,107739108		
76_NTD_140407-005_LHa_SH_rb_7.D	adult residual body	15,6078149			0,099524503		
78_NTD_140407-005_LHa_SH_rb_9.D	adult residual body	8,254580867			0,109382331		
80_NTD_140407-005_LHa_SH_rb_14.D	adult residual body	4,234344702			0,107532012		
09_NTD_140407-05_L_Ha_SH_3_rb.D	adult residual body	5,427142941			0,085694056		

HSAB (honey syrup-Sitotroga eggs-antibiotics diet)(feeding larvae to adult) data file		IPMP pg/mg FW	average IPMP pg/mg FW	Stdev IPMP pg/mg FW	IPMP pg/sample	average IPMP pg/sample	Stdev IPMP pg/sample
49_NTD_140407-005_LHa_AB_1.D	adult	2,7745246	9,210703099	9,618452037	0,075602499	0,099206734	0,024641157
50_NTD_140407-005_LHa_AB_2.D	adult	3,623408417			0,098086936		
51_NTD_140407-005_LHa_AB_3.D	adult	6,848121304			0,083768337		
52_NTD_140407-005_LHa_AB_4.D	adult	6,699217687			0,098971969		
53_NTD_140407-005_LHa_AB_5.D	adult	26,10824349			0,13960393		
54_NTD_140407-005_LHa_AB_gut_4.D	adult gut	11,18854793	2,566317984	4,833730155	0,039706246	0,034263405	0,020529315
57_NTD_140407-005_LHa_AB_gut_8.D	adult gut	0,99065251			0,043116634		
59_NTD_140407-005_LHa_AB_gut_10.D	adult gut	0			0		
61_NTD_140407-005_LHa_AB_gut_13.D	adult gut	0,248975549			0,054358423		
63_NTD_140407-005_LHa_AB_gut_16.D	adult gut	0,403413929			0,034135724		
55_NTD_140407-005_LHa_AB_rb_4.D	adult residual body	2,938576532	2,712513347	1,266330252	0,09541272	0,108013108	0,029083663
58_NTD_140407-005_LHa_AB_rb_8.D	adult residual body	2,379651078			0,092386979		
60_NTD_140407-005_LHa_AB_rb_10.D	adult residual body	1,115342021			0,158107857		
62_NTD_140407-005_LHa_AB_rb_13.D	adult residual body	2,503896468			0,107846965		
64_NTD_140407-005_LHa_AB_rb_16.D	adult residual body	4,625100633			0,086311017		

Data files for SBMP, IPMP, and IBMP content during development of *H. axyridis* feeding on HS and HSAB diet

- **IBMP**

HS (honey syrup-Sitotroga eggs diet) (feeding L1-L4)		IBMP	average IBMP	Stdev IBMP	IBMP	average IBMP	Stdev IBMP
data file		pg/mg FW	pg/mg FW	pg/mg FW	pg/sample	pg/sample	pg/sample
12_NTD_140407-005_HaL4_SH_1.D	L4	0,36289397	0,1166578	0,167496859	11,66704114	3,616988892	5,289052106
13_NTD_140407-005_HaL4_SH_2.D	L4	0,220395032			6,417903326		
15_NTD_140407-005_HaL4_SH_3.D	L4	0			0		
16_NTD_140407-005_HaL4_SH_4.D	L4	0			0		
17_NTD_140407-005_HaL4_SH_5.D	L4	0			0		
18_NTD_140407-005_HaL4_SH_gut_1.D	L4 gut	0	0	0	0	0	0
20_NTD_140407-005_HaL4_SH_gut_5.D	L4 gut	0			0		
22_NTD_140407-005_HaL4_SH_gut_9.D	L4 gut	0			0		
24_NTD_140407-005_HaL4_SH_gut_13.D	L4 gut	0			0		
26_NTD_140407-005_HaL4_SH_gut_16.D	L4 gut	0			0		
19_NTD_140407-005_HaL4_SH_rb_1.D	L4 residual body	0	0	0	0	0	0
21_NTD_140407-005_HaL4_SH_rb_5.D	L4 residual body	0			0		
23_NTD_140407-005_HaL4_SH_rb_9.D	L4 residual body	0			0		
25_NTD_140407-005_HaL4_SH_rb_13.D	L4 residual body	0			0		
28_NTD_140407-005_HaL4_SH_rb_16.D	L4 residual body	0			0		

HSAB (honey syrup-Sitotroga eggs-antibiotics diet) (feeding L1-L4)		IBMP	average IBMP	Stdev IBMP	IBMP	average IBMP	Stdev IMMP
data file		pg/mg FW	pg/mg FW	pg/mg FW	pg/sample	pg/sample	pg/sample
33_NTD_140407-005_HaL4_AB_1.D	L4	0	0	0	0	0	0
35_NTD_140407-005_HaL4_AB_2.D	L4	0			0		
36_NTD_140407-005_HaL4_AB_3.D	L4	0			0		
37_NTD_140407-005_HaL4_AB_4.D	L4	0			0		
38_NTD_140407-005_HaL4_AB_5.D	L4	0			0		
39_NTD_140407-005_HaL4_AB_gut_2.D	L4 gut	0	0	0	0	0	0
41_NTD_140407-005_HaL4_AB_gut_5.D	L4 gut	0			0		
43_NTD_140407-005_HaL4_AB_gut_8.D	L4 gut	0			0		
46_NTD_140407-005_HaL4_AB_gut_12.D	L4 gut	0			0		
47_NTD_140407-005_HaL4_AB_gut_16.D	L4 gut	0			0		
40_NTD_140407-005_HaL4_AB_rb_2.D	L4 residual body	0	0	0	0	0	0
42_NTD_140407-005_HaL4_AB_rb_5.D	L4 residual body	0			0		
44_NTD_140407-005_HaL4_AB_rb_8.D	L4 residual body	0			0		
46_NTD_140407-005_HaL4_AB_rb_12.D	L4 residual body	0			0		
48_NTD_140407-005_HaL4_AB_rb_16.D	L4 residual body	0			0		

Data files for SBMP, IPMP, and IBMP content during development of *H. axyridis* feeding on HS and HSAB diet

- **IBMP**

HS (honey syrup-Sitotroga eggs diet (feeding larvae to adults))		IBMP	average IBMP	Stdev IBMP	IBMP	average IBMP	Stdev IBMP
data file		pg/mg FW	pg/mg FW	pg/mg FW	pg/sample	pg/sample	pg/sample
65_NTD_140407-005_LHa_SH_1.D	adult	0	0,618386225	1,245689939	0	17,03817225	33,56143395
66_NTD_140407-005_LHa_SH_2.D	adult	0			0		
67_NTD_140407-005_LHa_SH_3.D	adult	0			0		
68_NTD_140407-005_LHa_SH_4.D	adult	0			0		
70_NTD_140407-005_LHa_SH_5.D	adult	0,552476562			16,41407865		
06_NTD_140407-05_L_Ha_SH_1.D	adult	3,604408792			97,03068467		
07_NTD_140407-05_L_Ha_SH_2.D	adult	0,790204448			22,86061469		
08_NTD_140407-05_L_Ha_SH_3.D	adult	0			0		
71_NTD_140407-005_LHa_SH_gut_1.D	adult gut	0	0,042053608	0,103009882	0	0,206062681	0,504748424
73_NTD_140407-005_LHa_SH_gut_4.D	adult gut	0			0		
75_NTD_140407-005_LHa_SH_gut_7.D	adult gut	0,25232165			1,236376087		
77_NTD_140407-005_LHa_SH_gut_9.D	adult gut	0			0		
79_NTD_140407-005_LHa_SH_gut_14.D	adult gut	0			0		
10_NTD_140407-05_L_Ha_SH_3_gut.D	adult gut	0			0		
72_NTD_140407-005_LHa_SH_rb_1.D	adult residual body	0,224477238	0,49097056	0,541813947	4,348124095	10,29213522	11,48956983
74_NTD_140407-005_LHa_SH_rb_4.D	adult residual body	0,809366141			14,62524617		
76_NTD_140407-005_LHa_SH_rb_7.D	adult residual body	1,448289973			31,60168721		
78_NTD_140407-005_LHa_SH_rb_9.D	adult residual body	0			0		
80_NTD_140407-005_LHa_SH_rb_14.D	adult residual body	0,189343993			4,673009756		
09_NTD_140407-05_L_Ha_SH_3_rb.D	adult residual body	0,274346017			6,504744074		

HSAB (honey syrup-Sitotroga eggs-antibiotics diet)(feeding larvae to adult)		IBMP	average IBMP	Stdev IBMP	IBMP	average IBMP	Stdev IMMP
data file		pg/mg FW	pg/mg FW	pg/mg FW	pg/sample	pg/sample	pg/sample
49_NTD_140407-005_LHa_AB_1.D	adult	0	0,087437703	0,195516647	0	2,390546794	5,345425134
50_NTD_140407-005_LHa_AB_2.D	adult	0,437188514			11,95273397		
51_NTD_140407-005_LHa_AB_3.D	adult	0			0		
52_NTD_140407-005_LHa_AB_4.D	adult	0			0		
53_NTD_140407-005_LHa_AB_5.D	adult	0			0		
54_NTD_140407-005_LHa_AB_gut_4.D	adult gut	0	0	0	0	0	0
57_NTD_140407-005_LHa_AB_gut_8.D	adult gut	0			0		
59_NTD_140407-005_LHa_AB_gut_10.D	adult gut	0			0		
61_NTD_140407-005_LHa_AB_gut_13.D	adult gut	0			0		
63_NTD_140407-005_LHa_AB_gut_16.D	adult gut	0			0		
55_NTD_140407-005_LHa_AB_rb_4.D	adult residual body	0	0	0	0	0	0
58_NTD_140407-005_LHa_AB_rb_8.D	adult residual body	0			0		
60_NTD_140407-005_LHa_AB_rb_10.D	adult residual body	0			0		
62_NTD_140407-005_LHa_AB_rb_13.D	adult residual body	0			0		
64_NTD_140407-005_LHa_AB_rb_16.D	adult residual body	0			0		
